# Deciphering the Symbiotic Significance of Quorum Sensing Systems of *Sinorhizobium fredii* HH103

**DOI:** 10.3390/microorganisms8010068

**Published:** 2020-01-02

**Authors:** Sebastián Acosta-Jurado, Cynthia Alías-Villegas, Andrés Almozara, M. Rosario Espuny, José-María Vinardell, Francisco Pérez-Montaño

**Affiliations:** Departamento de Microbiología, Facultad de Biología, Universidad de Sevilla, 41012 Sevilla, Spain; sacosta@us.es (S.A.-J.); calias@us.es (C.A.-V.); andres.almozara@gmail.com (A.A.); espuny@us.es (M.R.E.); jvinar@us.es (J.-M.V.)

**Keywords:** quorum sensing, *Sinorhizobium fredii* HH103, AHL, rhizobia, LuxI-type proteins, LuxR-type proteins, symbiosis, nodulation, legumes

## Abstract

Quorum sensing (QS) is a bacterial cell-to-cell signaling mechanism that collectively regulates and synchronizes behaviors by means of small diffusible chemical molecules. In rhizobia, QS systems usually relies on the synthesis and detection of *N*-acyl-homoserine lactones (AHLs). In the model bacterium *Sinorhizobium meliloti* functions regulated by the QS systems TraI-TraR and SinI-SinR(-ExpR) include plasmid transfer, production of surface polysaccharides, motility, growth rate and nodulation. These systems are also present in other bacteria of the *Sinorhizobium* genus, with variations at the species and strain level. In *Sinorhizobium fredii* NGR234 phenotypes regulated by QS are plasmid transfer, growth rate, sedimentation, motility, biofilm formation, EPS production and copy number of the symbiotic plasmid (pSym). The analysis of the *S. fredii* HH103 genomes reveal also the presence of both QS systems. In this manuscript we characterized the QS systems of *S. fredii* HH103, determining that both TraI and SinI AHL-synthases proteins are responsible of the production of short- and long-chain AHLs, respectively, at very low and not physiological concentrations. Interestingly, the main HH103 *luxR*-type genes, *expR* and *traR*, are split into two ORFs, suggesting that in *S. fredii* HH103 the corresponding carboxy-terminal proteins, which contain the DNA-binding motives, may control target genes in an AHL-independent manner. The presence of a split *traR* gene is common in other *S. fredii* strains.

## 1. Introduction

The rhizobium–legume symbiosis is one of the best studied model systems of mutualistic interactions between bacteria and eukaryotic hosts. This symbiosis is initiated by a complex and evolved molecular exchange between both symbionts that culminate in the formation of nitrogen-fixing plant root nodules [1,2,3]. The infection is initiated by the exudation of plant-produced *nod* gene-inducing flavonoids [4,5,6]. These polyphenolic compounds are perceived by bacterial transcriptional regulators, i.e., NodD, that in turns induce expression of genes responsible for the synthesis of Nod factors (NF), the nodulation (*nod*) genes [4,7]. NF are released by rhizobia and recognized specifically by susceptible legumes, which triggers both root hair curling and cortical cell division [8]. Deformed root hairs entrap rhizobia that enter into the root tissue through infection threads, reach the cortex, multiply and colonize the intracellular spaces in root nodules, where nitrogen fixation takes place [1,8]. The symbiotic process is often very specific and most rhizobia establish symbiosis with a small number of legume hosts. However, some rhizobia have evolved mechanisms that allow them to nodulate a larger variety of legume plants [9,10]. *Sinorhizobium* (=*Ensifer*) *fredii* is a rhizobial species that has an extremely broad host range (more than 100 genera of legumes are nodulated) that includes plants forming determinate and indeterminate nodules, such as *Glycine max* (soybean) and *Glycyrrhiza uralensis*, respectively [11]. Nodulation ability of the three most studied *S. fredii* strains, NGR234, USDA257 and HH103 [12,13,14], is explained in part because plant flavonoids, in addition to NFs, regulate additional bacterial symbiotic-traits: Secretion of proteins through a type 3 secretion system (T3SS), exopolysaccharide (EPS) production, formation of biofilms and functioning of quorum sensing (QS) systems [15,16,17].

QS is a cell-to-cell signaling mechanism that allows bacteria to collectively modify and synchronize behaviors, some of which being important for the interaction with eukaryotic hosts, by means of small diffusible chemical molecules. In rhizobia, QS systems usually relies on the synthesis and detection of *N*-acyl-homoserine lactones (AHLs). LuxI-type synthases produce these molecules and the corresponding LuxR-type receptors regulate target gene transcription in the presence of cognate AHLs. In the model bacterium *S. meliloti*, functions regulated by QS include plasmid transfer, production of surface polysaccharides, surface translocation, motility, growth rate, and nodulation [18,19,20,21]. The QS systems TraI-TraR and SinI-SinR(-ExpR) described for *S. meliloti* are present in other species of *Sinorhizobium*, with variations at the species and strain levels [18,22]. In *S. fredii* NGR234, phenotypes regulated by their homologous QS systems, *tra* and *ngr* respectively, have been widely characterized: Plasmid transfer, growth rate, sedimentation, motility, biofilm formation, EPS production, and the copy number of the symbiotic plasmid (pSym). Some of these phenotypes are involved in the symbiotic performance with legume hosts whereas others seem to play a role during free-living conditions [22]. The analysis of the *S. fredii* HH103 and USDA257 genomes reveal also the presence of *tra* and *sin*/*ngr* QS systems [13,14]. However, no reports dealing with these QS systems have been published so far for any *S. fredii* strain other than NGR234.

In this work, we characterized the QS systems of *S. fredii* HH103 and studied whether they have a role in the regulation of important symbiotic molecules/traits, such as growth rate, surface polysaccharides production, biofilm formation, or motility. We also assessed the influence of these systems on symbiosis with two legume hosts, *Glycine max* and *Glycyrrhiza uralensis*. We determined that both *traI* and *sinI* AHL-synthases genes are producing short- and long-chain AHLs at not physiological concentrations, being not relevant for any symbiotic trait analyzed. Interestingly, the main HH103 *luxR*-type genes, *traR* and *expR*, are truncated, although putative proteins containing the original carboxy-terminal part of TraR and ExpR (which harbor the DNA-binding motif), might control target genes in an AHL-independent manner. We hypothesize that this fact could be an evolutionary strategy to enhance symbiosis with a broader legume host-range.

## 2. Materials and Methods

### 2.1. Bacterial Strains and Plasmids

Bacterial strains and plasmids used in this work are listed in Table 1. Rhizobial strains used in this study were grown at 28 °C on tryptone yeast (TY) medium [23] or yeast extract mannitol (YM) medium [24], supplemented with genistein when necessary. Genistein was dissolved in ethanol and used at 1 μg mL^−1^ to give a final concentration of 3.7 μM. *Escherichia coli* strains were cultured on LB medium [25] at 37 °C. *Agrobacterium tumefaciens* NT1 (pZLR4) and *A. tumefaciens* GMI9023 (pMUS248) were grown at 28 °C in YM and TY, respectively. When required, the media were supplemented with the appropriate antibiotics as previously described [26]. Commercial AHLs were dissolved in methanol and used at different concentrations. Flavonoids and AHLs were purchased from Fluka (Sigma-Aldrich, St. Louis, MO, USA). The growth curves were obtained with a Sinergy HT microplate reader (BioTek, Winooski, VT, USA) and growing the bacteria for 72 h at 28 °C with continuous orbital shaking. Measurements were made every 2 h.

Plasmids were transferred from *E. coli* to HH103 by conjugation as described by Simon [34] using plasmid pRK2013 as helper. Recombinant DNA techniques were performed according to the general protocols of Sambrook et al. [35]. PCR amplifications were performed as previously described [36]. Primer pairs used for the amplification of the *S. fredii* HH103 genes are summarized in Table 2. 

The complete HH103 *traI* and *sinI* genes were amplified using specific primers and the resulting DNA fragments were cloned into pGEM-T Easy (Promega, Madison, WI, USA) obtaining plasmids pMUS997 and pMUS1079, respectively. These plasmids were digested with suitable restriction enzymes and fragments containing full *traI* and *sinI* ORFs were cloned into suicide plasmid pK18mob, previously digested with the same enzymes, obtaining plasmids pMUS989 and pMUS1083, respectively. Then, the plasmids pHP45Ω and pAB2001 were digested with restriction enzymes to extract DNA fragments carrying the interposon Ω or the cassette *lacZ*-Gm^R^, respectively. These DNA fragments were cloned in each gene into an unique restriction site of pMUS989 and pMUS1083, respectively, to obtain the pMUS1006 (pK18mob::*traI*::Ω) and pMUS1087 (pK18mob::*sinI*::*lacZ-*Gm^R^) plasmids. These plasmids were employed for the homogenotization of the mutated version of the *traI* and *sinI* genes in *S. fredii* HH103 generating the mutant strains in each of these genes as well as a double mutant affected in both genes. Double recombination events were confirmed by southern blot (data not shown). For hybridization, DNA was blotted to Hybond-N nylon membranes (Amersham, Amersham, UK), and the DigDNA method of Roche (Basel, Switzerland) was employed according to the manufacturer’s instructions.

### 2.2. Well Diffusion Assay and Thin Layer Chromatography Analysis

*A. tumefaciens* NT1 (pZRL4) was used for the detection of AHLs (acyl chains ranging from C4 to C18) from supernatants of the parental strain grown at OD_600_ 1.2 in well diffusion assays in Petri dishes as described by McClean et al. and Cha et al. [28,37]. Supernatants of the rhizobial strains grown at different OD_600_ (0.3, 0.6, 0.9, and 1.2) in 5 mL of YM medium were extracted with dichloromethane, evaporated to dryness, resuspended in 5 µL of methanol and analyzed by thin layer chromatography (HPTLC plates RP-18 F254s 1.13724 and 1.05559, Merck, Darmstadt, Germany) using methanol:water (60:40 *v*/*v*) as eluent, dried and developed with *A. tumefaciens* NT1 (pZLR4).

### 2.3. HPLC and Mass Spectrometry Analysis

Rhizobia were grown in 500 mL of YM medium for 6 days at 28 °C with shaking. Cultures were centrifuged and supernatants were extracted with dichloromethane and the organic layer was evaporated. The extracts were dissolved in 1 mL of methanol:water (1:1 *v*/*v*) containing 0.1% (*v*/*v*) formic acid, microfiltered (0.2 µm), and 20 mL injected onto an HPLC system equipped with a Tracer Hypersyl ODS column (250 × 4.6 mm, 5 µm particle size) (Teknokroma, Sant Cugat del Vallès, Spain). Elutions were carried out at room temperature with a flow rate of 400 µL min^−1^ using a gradient of water and methanol, both acidified with 0.1% formic acid [38]. HPLC studies were performed on a Perkin Elmer Series 200 HPLC system (Waltham, Waltham, MA, USA) coupled to a mass spectrometer.

Methods using high pressure liquid chromatography and tandem mass spectrometry (HPLC-MS/MS) have been applied to profile the bacterial QS molecules from different bacteria [38,39,40,41]. Multiple-Reaction ion Monitoring (MRM) is a tandem MS/MS method in which user-selected specific ions are transmitted through the first analyzer (a quadrupole, Q1) and user-selected specific fragments arising from collision induced decomposition (CID) in Q2 are measured by the second analyzer (Q3). This technique has shown to be very suitable for the identification and study of components in very complex mixtures. Since both precursor (usually pseudomolecular ion, [M + H]^+^) and product ions must be indicated before carrying out the analysis, the compound to identify must be known and has been well-characterized previously before this type of experiment is undertaken. The AHL family shows pseudomolecular peaks at [M + H]^+^ when electrospray is performed in positive mode (precursor ion), and the CID fragmentation generates a common product ion at m/z 102, which formally corresponds to this ring plus the nitrogen atom as NH_3_^+^, together with the product ions corresponding to [M + H − 101]^+^.

A second IDA method, called “ion precursor”, was also used in the analysis. This method was set to register those compounds that generate fragments at m/z 102 in Q3. This technique results complementary to MRM, as it can detect those compounds which are not included in the MRM Q1/Q3 list, such as adducts of AHL with ammonium or solvent molecules [42], although the solvents were acidified and the declustering potential was optimized to minimize the formation of this kind of adducts [43]. Besides IDA methods, enhanced product ion (EPI) spectra were also recorded of the two more intense peaks (above 4000 counts per second) to verify the structure of the detected AHL by comparison with mass spectra of standards. 

All MS experiments were conducted on a 2000 QTRAP hybrid triple-quadrupole-linear trap mass spectrometer (Applied Biosystem, San Francisco, CA, USA) equipped with a Turbo Ion source used in positive ion electrospray mode. Mass spectrometric conditions were optimized by infusing solutions of standards dissolved in methanol (100 mg ml^–1^) at a flow of 10–100 mL min^–1^: C6-HSL, 3-oxo-C6-HSL, C8-HSL, 3-oxo-C8-HSL, C10-HSL, 3-oxo-C10-HSL, C12-HSL, 3-oxo-C12-HSL, 3-OH-C12-HSL, C14-HSL, 3-oxo-C14-HSL, and 3-OH-C14-HSL. The probe capillary voltage was optimized at 5500 V. Desolvation temperature was set to 50 °C. Pressures of curtain, nebulizing, and turbo spray gases were set to 35, 20, and 0 (arbitrary units), respectively. Nitrogen was used for CID. Ions were scanned from m/z 150 to m/z 500 at a scan rate of 4000 Th s^−1^.

### 2.4. EPS Production and Analysis of Lipopolysaccharide (LPS) and K-Antigens Polysaccharide (KPS)

For analysis of EPS production in solid media, 20 μL droplets of YM-grown early log (OD_600_ 0.4) cultures were placed onto YM plates supplemented with genistein when necessary, incubated at 28 °C for 96 h and photographed. LPS extraction from bacterial cultures grown on solid TY medium, separation on SDS-PAGE gels and silver staining were carried out as described by Buendía-Clavería et al. [44]. K-antigen capsular polysaccharides (KPS) were extracted from bacterial cultures grown on solid TY medium and analyzed by PAGE as described by Hidalgo and colleagues [45].

### 2.5. Motility Assays

Swimming was examined on plates prepared with Bromfield medium (BM) [46] containing 0.3% agar, supplemented with genistein when necessary, and inoculated with 3 µL aliquots of rhizobial cultures grown in TY (OD_600_ 1.0). The migration zone was determined as the colony diameter (mm) after 24 h, 48 h, and 7 days of incubation. Each experiment was performed three times. The swimming motility of each strain were compared to that of the parental strain by the Mann–Whitney nonparametric test. 

### 2.6. Biofilm Formation Assays

The biofilm formation assay was based on the method described by O’Toole and Kolter [47] with modifications [48]. Cultures were grown in 5 mL of low-phosphate MGM medium supplemented with genistein when necessary [47], diluted to an OD_600_ of 0.2 and inoculated with 100 µL aliquots and placed on polystyrene microtiter plates, U form (Deltalab S.L., Rubí, Spain). The plates were inverted and incubated at 28 °C for 7 days with gentle rocking. Cell growth was analyzed by measuring OD_600_ using a microtiter reader Synergy HT (Biotek, Winooski, VT, USA). The culture in each well was removed carefully; the wells were dried, washed three times with 0.9% NaCl and dried again. Biofilms in each well were stained with 100 µL of 0.1% crystal violet for 20 min, then washed with water three times and dried again. Finally, 100 µL of 96% ethanol were added to each well and the OD_570_ was measured. Numbers provided are the average value ± standard deviation of three independent experiments with eight technical replicates each. The biofilm produced by each strain were compared to that of the parental strain by the Mann–Whitney nonparametric test.

### 2.7. Nodulation Assays

For the evaluation of the symbiotic phenotypes, the wild-type and derivative mutant strains of HH103 were grown in YM medium. Surface-sterilized seeds of *G. max* (determinate nodules) and *G. uralensis* (indeterminate nodules) were pre-germinated and placed in sterilized Leonard jars and test tubes respectively, containing Fårhaeus N-free solution [24]. Germinated seeds were then inoculated with 1 mL of bacterial culture in a concentration approx. 10^9^ cells mL^−1^. Growth conditions were 16 h at 26 °C in the light and 8 h and 18 °C in the dark, with 70% of humidity. Nodulation parameters were evaluated after 6 weeks for *G. max* and after 8 weeks for *G. uralensis*. Shoots were dried at 70 °C for 48 h and weighed. Nodulation experiments were performed three times with six replicates for each treatment. For the different parameters analyzed, the values of each treatment were compared to those of *S. fredii* HH103 by using the Mann–Whitney non-parametrical test.

### 2.8. Protein Alignment

LuxR-type proteins were aligned using the ClustalW program and manipulated with Boxshade at EMBnet. 

### 2.9. RNA-seq Data Accession Number and Gene Transcript Assignment 

The RNA-seq data obtained from *S. fredii* HH103 grown in YM medium in the absence of genistein and discussed in this publication are deposited in the Sequence Read Archive of NCBI (BioProject database) under the BioProject ID PRJNA313151. To obtain the number of transcripts assigned to each gene, the initial whole transcriptome paired-end reads obtained from sequencing [49] were mapped against the latest version of the *S. fredii* HH103 genome (http://www.ncbi.nlm.nih.gov/assembly/GCF_000283895.1/) using the Life Technologies mapping algorithm version 1.3 (http://www.lifetechnologies.com/). Low-quality reads were eliminated using Picard Tools software version 1.83, remaining only high quality reads. Transcriptomic ranking for each gene (%) was established sorting all *S. fredii* HH103 genes according to the number of transcripts assigned for each and implementing the following formula: (1 − (transcriptomic relative position/total gene number)) × 100.

### 2.10. Calculation of pSym Conjugation Frequency

Conjugation of the pSym of *S. fredii* HH103 and its *sinI* and/or *traI* mutants to *A. tumefaciens* GMI9023 carrying plasmid pMUS248 were carried out by biparental mating. Plasmid pMUS248 contains a tetracycline-resistance gene under the transcriptional control of the *nodA* promoter of *Rhizobium leguminosarum* bv. *viciae*, thus conferring Tc^R^ in the presence of a NodD protein and appropriate flavonoids [29]. Donor strains were cultured on TY, whereas GMI9023 (pMUS248) was cultured on TY supplemented with kanamycin (25 µg/mL) for maintaining of plasmid pMUS248. Briefly, cells of 1 mL of stationary phase cultures of donor and recipient (after washing with TY for eliminating Km) were collected, mixed, resuspended in 100 µL of TY, deposited onto a TY plate, and incubated at 28 ºC for 24 h. The developed bacterial biomass was then resuspended in 1 mL of TY and plated at different dilutions onto TY plates supplemented with chloramphenicol (4 µg/mL) and kanamycin (25 µg/mL) for selecting the recipient and TY plates containing chloramphenicol (4 µg/mL), kanamycin (25 µg/mL), tetracycline (15 µg/mL), and genistein (1 µg/mL) for selecting GMI9023 (pMUS248) transconjugants that had received the pSym of the donor strain. Genistein is an effective *nod* gene inducer for *S. fredii* HH103 [29]; thus, those transconjugants of GMI9023 (pMUS248) that had received a pSym plasmid containing the *nodD1* gene of HH103 are able to resist Tc at 15 µg/mL when grown in the presence of genistein. As a control, cultures of GMI9023 (pMUS248) were unable to grow in the presence of tetracycline at such concentration. The frequency of conjugation of the pSym for each donor strain was calculated as the ratio between the numbers of transconjugants cells/mL and recipient cells/mL. Numbers provided are the average value ± standard deviation of four independent experiments. The presence of the pSym of the donor strain in 40 different transconjugants was confirmed by PCR using specific primers for the *S. fredii* HH103 *nodD1* gene (Table 2), which allows amplification of a 171-bp internal fragment of that gene.

### 2.11. Quantification of Plasmid Copy Number

gDNA extraction and quantitative PCR were performed as previously described [50]. Total DNA was isolated using GenElute^TM^ Bacterial Genomic DNA Kit (Sigma-Aldrich, St. Louis, MO, USA). Samples were analyzed with primer sets (Table 2) specifically targeting four plasmid genes (*nodA*, *syrM*, psfHH103d_306, and psfHH103d_373) and four chromosomal genes (*ligE*, *ftsZ1*, *flgJ,* and *nolR*). Quantitative PCR was performed using a LightCycler 480 (Roche, Basel, Switzerland) and SYBR® Green Master Mix (Biorad, Hercules, CA, USA) with the following conditions: 95 °C, 10 min; 95 °C, 30 s; 50 °C, 30 s; 72 °C, 20 s; forty cycles, followed by the melting curve profile from 60 to 95 °C to verify the specificity of the reaction. Plasmid copy number was defined as the plasmid DNA: chromosome ratio, using the formula 2^-ΔCT^, where ΔCT is the difference in average threshold cycles (Ct) between plasmid and chromosomal genes. Numbers provided are the average value ± standard deviation of three independent experiments (each one using different cultures and DNA extractions).

## 3. Results

### 3.1. S. fredii HH103 Produces Short- and Long-Chain AHLs at not Physiological Concentrations by Means of TraI and SinI, Respectively

**Figure 1:**
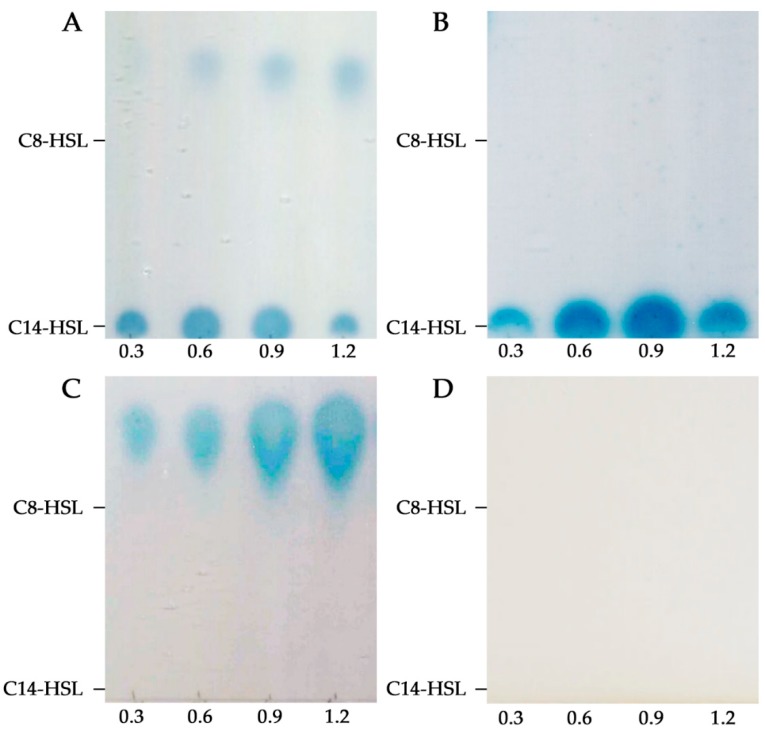
thin layer chromatography (TLC) analysis of N-acyl-homoserine lactones (AHLs) produced (100-fold) by *S. fredii* HH103 strains at four different OD600 (0.3, 0.6, 0.9 and 1.2) in YM medium. Migration of two different AHL standards (C8-HSL and C14-HSL) is shown on the left of each panel. (**A**) HH103. (**B**) HH103 *traI*. (**C**) HH103 *sinI*. (**D**) HH103 *traI sinI*.

The analysis of the *S. fredii* HH103 genome reveals the presence of two genes (psfHH103d_478 and SFHH103_01571) encoding for the LuxI-type synthases TraI (CEO91679.1) and SinI (CCE96069.1) [14]. Supernatants from *S. fredii* HH103 cultures grown at stationary phase (OD_600_ 1.2) were first assayed for AHLs production in well diffusion assays by using the biosensor *A. tumefaciens* NT1 (pZRL4). Unexpectedly, these experiments showed that this rhizobial strain does not produce detectable AHLs at physiological concentrations (Figure S1). In order to concentrate 100-fold the putative AHLs present in HH103 supernatants, 5 mL of wild-type cultures grown at four different OD_600_ (0.3, 0.6, 0.9, and 1.2) were extracted with dichloromethane, evaporated, resuspended in 5 µL of methanol and analyzed by thin layer chromatography (TLC) using the same biosensor strain. By using this methodology, two spots corresponding to short- and long-chain AHLs could be detected (Figure 1a). However, the two AHL-types detected showed different kinetics of production: Short-chain AHLs accumulation increased according to bacterial growth while long-chain AHLs accumulation reached its highest value at late exponential phase and then decreased. Mass spectrometry analyses unequivocally identified the C8-HSL, 3-oxo-C8-HSL, C12-HSL, C14-HSL, and 3-oxo-C14-HSL in supernatants of *S. fredii* HH103 cultures (Table 3), which is consistent with the occurrence of both short- (acyl chains between C4 and C8) and long-chain (acyl chains between C10 and C16) AHLs in TLC assays. 

To further investigate the role of each *luxI*-type gene in the production of AHLs, single and double mutants in the *traI* and *sinI* genes were constructed and analyzed for the production of AHLs. TLC and mass spectrometry assays showed that the HH103 *traI* only produced the long-chain AHLs (Figure 1b), indicating that TraI is involved in the synthesis of at least the C8-HSL and 3-oxo-C8-HSL. In contrast, when the *sinI* mutant supernatants were assayed, only the short-chain AHLs were detected and identified (Figure 1c), demonstrating that SinI produces at least the C12-HSL, C14-HSL, and 3-oxo-C14-HSL. As expected, no AHLs were detected in supernatants of the double mutant (Figure 1c), which points out that SinI and TraI might be the only LuxI-type synthases present in the genome of *S. fredii* HH103. 

### 3.2. S. fredii HH103 AHLs Do Not Regulate Symbiotically Important Traits and Have No Influence on the Symbiotic Performance with G. max and G. uralensis

As commented above, the QS systems of *S. meliloti* and *S. fredii* NGR234 are controlling some important traits such as growth rate, surface polysaccharide production, motility, or biofilm formation that, to a greater or lesser extent, are related with an optimal symbiotic performance [22]. In order to decipher the symbiotic relevance of QS systems of *S. fredii* HH103, these nodulation-related traits were analyzed in the wild-type and mutant strains. First of all, analyses of their growth in TY and YM media indicated that that inactivation of *sinI* and/or *traI* has not impact in the bacterial growth rate, indicating that any difference that could further be observed among strains is not due to an effect on bacterial growth (Figure S2). 

In some *S. meliloti* strains the presence of AHLs is necessary for the synthesis of symbiotically important exopolysaccharides (EPS) [22,51]. In this work we have analyzed three *S. fredii* HH103 important polysaccharides in symbiosis: EPS, capsular polysaccharides (KPS or K-antigens), and lipopolysaccharides (LPS) [52]. The *traI*, *sinI* and *traI/sinI* mutant strains were investigated for the production of KPS or LPS by growing them in TY medium and analyzing by polyacrylamide gel electrophoresis (PAGE) cell extracts enriched in these polysaccharides. KPS were visualized by a treatment with Alcian Blue and silver staining. No differences were detected among KPS profiles in any of the assayed strains (Figure S3a). LPS profiles of these mutants grown in TY medium were also analyzed by PAGE assays performed in the presence of SDS followed by a silver staining. The LPS electrophoretic profile of the different mutants was unaltered in comparison with that of HH103 (Figure S3b). The study of the EPS was performed analyzing the mucoidy in YMA medium, supplemented or not with genistein. All bacterial strains presented mucoid phenotypes in the absence of inducing-flavonoid, which is indicative of EPS production (Figure S3c). In contrast, in the presence of genistein, all strains displayed rough appearance, consequence of EPS repression mediated by NodD1 and flavonoids in this strain [17]. Altogether, these results indicate that AHLs are not regulating the synthesis of these important symbiotic polysaccharides in *S. fredii* HH103.

Besides to activate EPS synthesis, the QS systems of *S. meliloti* also promote repression of flagella synthesis at high population densities, which is related with a lower bacterial motility [22,50]. In order to determine the role of AHL perception in this process, the swimming motility of the HH103 *traI*, *sinI,* and *traI/sinI* mutants in Bromfield medium in the absence or presence of genistein was assayed. No differences were detected in any of the genetic backgrounds analyzed in both conditions after 24 h, 48 h, or 7 days (Figure S4a). On the other hand, another important trait regulated by QS in some strains of *S. fredii* is the formation of symbiotic biofilms, required for a successful colonization of legume root, which is important for the nodulation process [53]. Biofilm formation was assessed by experiments of adhesion to polystyrene surface of the different of *S. fredii* HH103 strains after 7 days of growing in low-phosphate MGM medium supplemented or not with genistein. No significant differences were detected among strains and conditions (Figure S4b). These results point out that AHLs produced by LuxI-type synthases of HH103 are neither involved in the control of swimming motility nor in that of biofilm formation.

Finally, the influence of the AHL production of HH103 in symbiotic performance was also investigated. As commented in introduction, *S. fredii* HH103 nodulates a broad range of legumes, including both determinate- and indeterminate-nodule forming plants. Thus, the symbiotic phenotype of HH103 *sinI* and/or *traI* mutants was investigated with *G. max* (determinate nodules) and *G. uralensis* (indeterminate nodules) plants (Table S1). None of the mutants showed any significant defect with any of the two legumes analyzed when compared to the wild-type strain. 

### 3.3. The traR and expR Genes of S. fredii HH103 Are Divided in Two ORFs 

**Figure 2:**
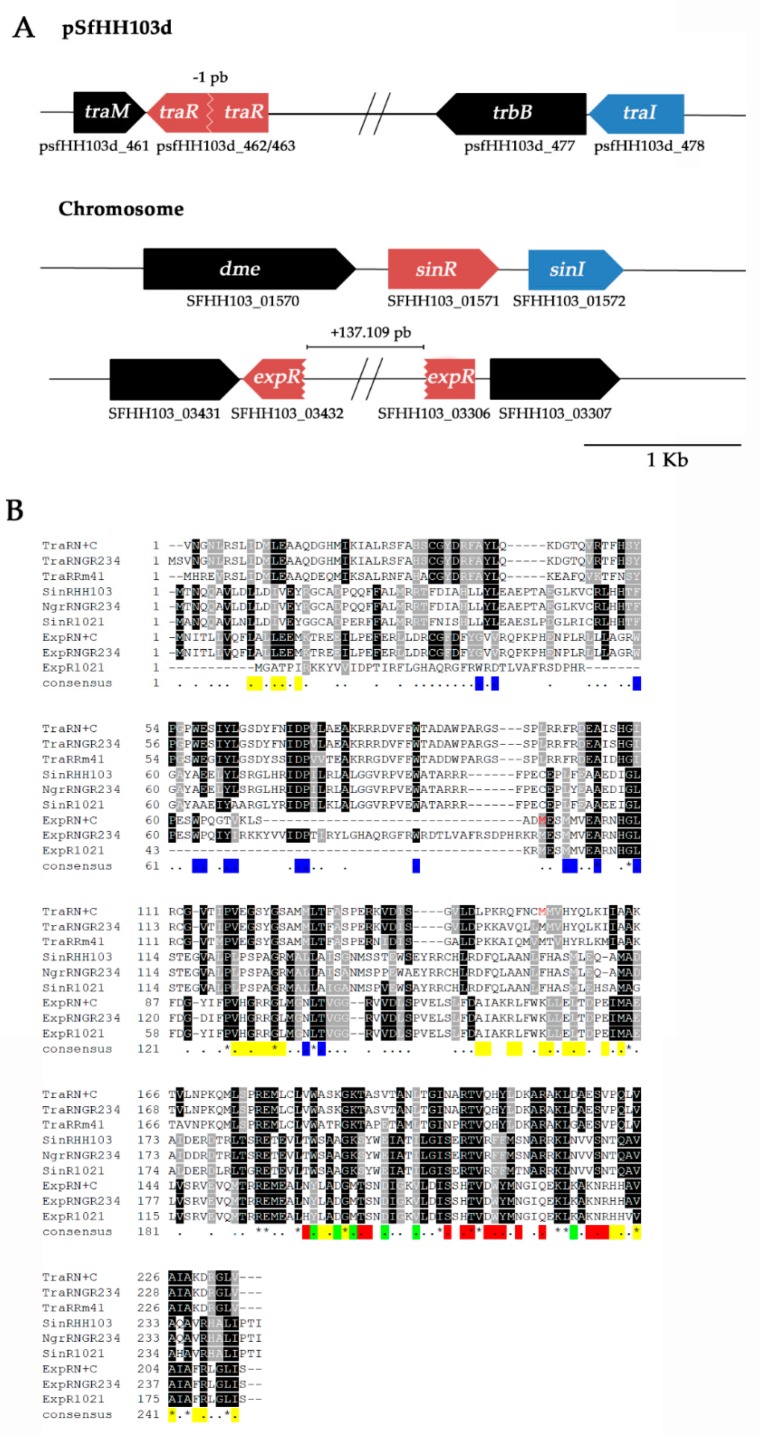
(**A**) Gene neighborhood of the *Sinorhizobium fredii* HH103 QS genes. In blue *luxI* homologous genes. In red: *luxR* homologous genes. Genomic distance between the psfHH103_463 and psfHH103_462 ORFs is −1 pb (these genes overlap in one nucleotide), whereas between SFHH103_03432 and SFHH103_03306 genes the distance is 137.109 pb. (**B**) Multiple sequence alignment of nine LuxR homologues (reconstructed TraR from *Sinorhizobium fredii* HH103, TraR from *S. fredii* NGR234, TraR from *Sinorhizobium meliloti* Rm41, SinR from *S. fredii* HH103, NgrR from *S. fredii* NGR234, SinR from *S. meliloti* 1021, reconstructed ExpR from *S. fredii* HH103, ExpR from *S. fredii* NGR234 and ExpR from *S. meliloti* 1021). Proteins were aligned using the ClustalW program and manipulated with Boxshade at EMBnet. Dark and gray boxes indicate identical and similar amino acids, respectively. First residue of the C-terminal domains of the reconstructed proteins are in red. Residues functions have been calculated according to those determined for the TraR protein of *A. tumefaciens* [59]: Residues interacting with AHL and with DNA fragments are marked in the consensus line with blue and red, respectively; residues interacting with RNA polymerase are indicated by green in the consensus line; residues in contact at the dimer interface are marked in yellow in the consensus line.

The low production of AHLs by strain HH103 as well as their irrelevance in the different traits analysed prompted us to investigate whether, in contrast to that described for *S. meliloti* and *S. fredii* NGR234, the *S. fredii* HH103 *tra* and *sin* QS systems are not functional. We have demonstrated that TraI and SinI are functional AHL synthases that account for the production of short- and long-chain AHLs, respectively, although at very low concentrations. Then, the question that raises is whether the other elements necessary for the correct functioning of the QS systems, the LuxR-type proteins, were active in *S. fredii* HH103. The genetic organization of the *tra* and *sin* QS system genes in the *S. fredii* HH103 genome are shown in Figure 2a. The analysis of the sequence of *traR* of *S. fredii* HH103 indicated that this gene has undergone a 2-bp deletion resulting in an early stop codon and in the formation of two ORFs overlapping in one nucleotide, psfHH103_463 and psfHH103_462, which code for 152- and 82-amino acid polypeptides (CEO91664.1 and CEO91663.1), respectively, and are 98.6% and 100% identical to the corresponding parts of the TraR protein of *S. fredii* NGR234 (236 amino acids) and 77.7% and 85.4% identical to the same parts of this protein of *S. meliloti* Rm41 (234 amino acids). In the other hand, the chromosomal *sinR* gene of HH103 (SFHH103_01570) codes for a complete 244 amino acids protein (CCE96068.1) that is 97.5% and 86.5% identical to the corresponding NgrR (*S. fredii* NGR234, 244 amino acids) and SinR (*S. meliloti* 1021, 245 amino acids) proteins, respectively. This LuxR-type transcription regulator controls *sinI* expression in an AHL-independent manner in *S. meliloti* [54,55,56]. Instead, the product of the *expR* gene, an orphan LuxR-type protein, is the major regulator of AHL-controlled genes in this bacterium [51,57,58]. Interestingly, the *expR* gene is also truncated and divided into two putative ORFs (SFHH103_03432 and SFHH103_03306) that are separated by nearly 140-kb in the genome of *S. fredii* HH103. These ORFs codes for 73- and 140-amino acid polypeptides (CCE97798.1 and CCE97923.1), respectively, and are 94.2% and 99.3% identical to the corresponding parts of the ExpR protein of *S. fredii* NGR234 (246 amino acids) and 91.3% and 98.5% identical to the same parts of this protein of *S. meliloti* 1021 (184 amino acids). Interestingly, sequence comparisons between full length TraR, SinR, and ExpR proteins from NGR234 and proteins coded by genes present in the genomes of the 13 *S. fredii* strains deposited in the public repository Integrated Microbial Genomes & Microbiomes of the Joint Genome Institute (JGI-IMG) showed that the presence of a truncated *traR* gene is a common phenomenon shared by nine *S. fredii* strains (Table S2), one of them (CCBAU83753) containing a truncated and a complete version of this gene. In addition, strain USDA205 does not contain an orthologue of *traR* in its genome. All the strains analyzed contain a complete *sinR* gene (Table S3) whereas *expR* is only truncated in the genomes of *S. fredii* HH103 and USDA257 (Table S4). 

### 3.4. The Terminal Parts of traR and expR Genes Code for Proteins That Conserve the DNA-binding Motives and the RNA Polymerase-Recruitment Residues 

LuxR-type proteins are composed of two functional domains: An amino-terminal (N-term) domain involved in AHL-binding and a carboxy-terminal (C-term) transcription regulation domain, which includes a helix–turn–helix (HTH) DNA-binding motif. In the presence of suitable AHLs, the LuxR-type proteins dimerize and interact with DNA recognizing specific sequences located in the regulatory regions of the target genes and recruiting the RNA polymerase to enhance the transcription of the target genes [59]. However, the deletion of the LuxR N-terminal domain results in a protein that is able to interact with DNA and to activate the transcription of target genes even in the absence of AHL [60]. Thus, for a more exhaustive analysis of the implication of the separation of the *luxR*-type genes in two ORFs, the two full LuxR-type proteins of *S. fredii* HH103 were reconstructed in silico by joining the two putative ORFs that would be forming the full *traR* and *expR* genes. Multiple sequence alignment of 9 LuxR-type proteins (including reconstructed proteins) of the *Sinorhizobium* genera and comparison with findings of previous studies with the TraR protein of *Agrobacterium tumefaciens* [59] allowed the identification of identical and similar amino acid residues; residues interacting with the AHLs, DNA and RNA polymerase; and residues in contact at the dimer interface of distinct LuxR-type proteins (Figure 2b). Interestingly, this alignment shows that for both genes of HH103 the two resulting ORFs respectively contain the full N-terminal (responsible for interaction with AHLs) and C-terminal (responsible for interaction with DNA) domains. This finding strengthens the possibility that the “second” ORF of both *traR* and *expR* may code for functional LuxR C-term domains that contains the HTH DNA-binding motive and RNA polymerase-recruitment residues (hereafter TraR C-term and ExpR C-term proteins). 

### 3.5. EPS Production Genes But Not Motility or Plasmid Transfer Genes Are Highly Expressed at High Population Densities

According to in silico analysis, the TraR C-term and ExpR C-term proteins maintain both the DNA-binding and the RNA-recruitment domains. So, could be QS-target genes being activated in an AHL independent manner in *S. fredii* HH103? To shed light on this hypothesis, RNA-seq data from *S. fredii* HH103 grown in YM medium in the absence of flavonoid obtained in a previous transcriptomic assay [49] and deposited in public repositories were analyzed in order to check the expression levels of putative target genes of both TraR C-term and ExpR C-term proteins. As commented above, in the *Sinorhizobium* genera both *tra* and *sin*/*ngr* systems regulated plasmid transfer, motility, and EPS production in an AHL-dependent manner [22]. Annotation of the *S. fredii* HH103 genome reveals the existence of different sets of genes coding for proteins involved in these important symbiotic traits: *tra* and *trb* genes code for plasmid conjugal transfer proteins; *fli*, *fla*, *flg*, *flh*, and *mot* products are involved in motility and chemotaxis; and proteins codified by *exo* genes are responsible for the synthesis of HH103 EPS [18]. The numbers of transcripts assigned to all these genes as well as their transcriptional ranking are shown in Table 4. In YM medium at stationary phase (OD_600_ 1.2) in the absence of inducing flavonoids, the transcripts number average assigned to the *tra* and *trb* genes of the symbiotic plasmid of *S. fredii* HH103 was 203, which corresponds to a transcriptional ranking of 30.7% (i.e., the 69.3% of HH103 genes present higher transcriptional rates). In the case of chemotaxis and motility genes, the average number of transcripts assigned to each gene was only 58, being their transcriptional ranking 17.3%. However, the *exo* genes accumulated an average of 5824 transcripts per gene, which indicate that 85.8% of the genes of the HH103 genome are less transcribed than genes involved in EPS production. These results indicate that, under the tested conditions, *S. fredii* HH103 is highly expressing *exo* genes, which would explain the mucoid phenotype observed in EPS production assays (Figure S2c). In contrast, both plasmid transfer and motility genes are not highly expressed under the same conditions, which, for the latter, is in accordance with results obtained in swimming motility experiments (Figure S3a). 

Finally, RNA-seq analysis for QS system genes strengthen previous findings, since the number of transcript assigned to *traI* and *sinI* were quite low (54 and 82, respectively) whereas both *traR* and *expR* C-term presented higher numbers of assigned transcripts in the tested conditions (541 and 450, respectively) (Table 4). 

### 3.6. S. fredii HH103 sinI and/or traI Mutants Show Similar Conjugation Rates of Their pSym Than the Wild-Type Strain

In *A. tumefaciens*, conjugal transfer of pTi is regulated by the *tra* QS system [61]. In this work we have studied whether the *tra* or the *sin* QS systems influenced the rate of conjugal transference of the *S. fredii* HH103 pSym. For this purpose, we estimated the conjugal transfer rate of the pSym of *S. fredii* HH103 and its *sinI* and/or *traI* mutant derivatives to *A. tumefaciens* GMI9023 carrying plasmid pMUS248 as described in Material and Methods. Plasmid pMUS248 contains a transcriptional fusion between a *nodA* promoter and a tetracycline resistant gene lacking its own promoter, thus conferring resistance to tetracycline in the presence of a NodD protein and an appropriate flavonoid [29]. GMI9023 (pMUS248) transconjugants carrying the pSym of either HH103 or its *sinI* and/or *traI* mutants become Tc^R^ in the presence of genistein, a *nod* gene inducer for *S. fredii* HH103 [29], thus allowing direct selection of these transconjugants. By using this approach, the conjugal rates of the pSym of HH103, HH103 *sinI*, HH103 *traI* and HH103 *sinI*/*traI* were estimated as 1.08 ± 0.28 × 10^−7^, 1.01 ± 0.30 × 10^−7^, 0.89 ± 0.41 × 10^−7^, and 0.84 ± 0.22 × 10^−7^, which indicate that neither the absence of *sinI* nor that of *traI* have a significant impact on the conjugation mobility of the HH103 pSym.

### 3.7. The Symbiotic Plasmid of S. fredii HH103 Is Present in about 3 Copies per Cell with Regard to the Chromosome

*S. fredii* HH103 produces short- and long-chain AHLs at very low concentrations. In *S. fredii* NGR234 the complete lack of QS molecules results in an elevated copy number of its symbiotic plasmid [62]. Therefore, copy number of the HH103 pSym on TY medium was determined based on analysis of DNA extracts in the presence and in the absence of genistein with four different chromosome primers pairs and four specific plasmid primer sets by quantitative PCR (Table 2). This analysis indicated that genes belonging to the symbiotic plasmid are ~2.5 ± 0.3-fold and ~3.3 ± 0.2-fold more abundant than those belonging to the chromosome in the absence and the presence of genistein respectively. Due to the fact that some cells within the population could have lost the plasmid during gDNA extraction, we cannot exclude the possibility that some cells could carry even more copies of this plasmid. These results indicate that the pSym is present in multicopy in *S. fredii* HH103.

## 4. Discussion

In the *Sinorhizobium* genera, five genes, *traI*, *traR*, *sinI*/*ngrI*, *sinR*/*ngrI*, and *expR*, are essential for QS regulation. In *S. meliloti* Rm41 and *S. fredii* NGR234, TraI is involved in the synthesis of a 3-oxo-C8-HSL, which is recognized by TraR, activating plasmid transfer [63,64]. Besides, to avoid plasmid conjugation when concentration of AHLs and population densities are low, another protein (TraM) competes with AHLs for binding to TraR, inhibiting the activation of plasmid conjugal transfer genes [65]. In *S. meliloti*, SinI is responsible for the production of diverse long-chain AHLs, ranging from C12-HSL to C18-HL with different substituents in the third carbon [54,55,57]. Upstream and adjacent to the chromosomal *sinI* gene is present *sinR*, which encodes a LuxR-type protein that regulates SinI expression in an AHL-independent manner [49,50,51]. Instead, the product of the *expR* gene, an orphan LuxR-type protein, is the major regulator of long-chain AHL-controlled gene expression in S. *meliloti* [51,57,58]. At moderated AHL concentrations, expression of *sinI* is strongly enhanced by ExpR in the typical positive feedback-type regulation. However, at very high AHL concentrations, ExpR is also repressing transcription of the *sinR* gene, which leads to negative feedback regulation of *sinI* [66]. In any case, in *S. meliloti* the presence of long-chain AHLs is necessary for the transcriptional regulation mediated by ExpR, including the two best-known functions controlled by this QS system: Activation of EPS synthesis and repression of flagella production [22]. In *S. fredii* NGR234, the homologous *ngr* QS system is also activating the expression of EPS-related genes but repressing chromosomal type IV genes, which in other bacteria are involved in twitching motility [22]. Besides, most of the ExpR-binding sites identified in *S. meliloti* are also present in the genome of NGR234 [56], suggesting that at least in some extend the SinI-SinR-ExpR regulatory network is conserved between *S. meliloti* and *S. fredii* NGR234. This regulatory network might operate in most of the *S. fredii* strains whose genomic sequences are available (thirteen) since all of them contains the three genes, with the exception of strains USDA257 and HH103, which carry a truncated version of *expR*.

**Figure 3:**
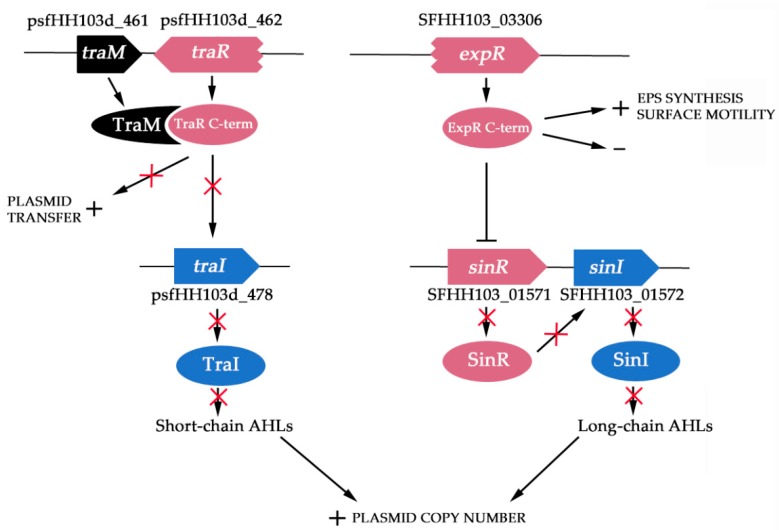
Hypothetic model of transcriptional regulation of quorum sensing (QS) in *Sinorhizobium fredii* HH103. Putative regulation of the *tra* and *sin* systems is shown. TraM binds to TraR C-term domain, what inhibits activation of plasmid conjugal transfer genes and synthesis of short-chain AHLs. ExpR C-term domain is repressing transcription of the *sinR* gene, which leads to negative feedback regulation of *sinI* and to inhibition of synthesis of long-chain AHLs. ExpR C-term domain is activating the synthesis of exopolysaccharides (EPS) and repressing surface motility in an AHL-independent manner. The absence of short- and long-chain AHLs increases the plasmid copy number.

In both *S. meliloti* and *S. fredii* NGR234, coordination of the expression of EPS production and motility could be facilitating the transition from free-living to symbiotic lifestyles. In the soil, when the bacterial population is low, chemotaxis and motility activation might be advantageous in search of an appropriate environment or host. However, once in the rhizosphere and due to the presence of root exudates, the rhizobial population density increases and QS might switch on to coordinate the repression of flagellar and/or pili production, which could interfere with proper progression of infection threads or activate plant defenses, and the activation of the production of EPS in order to facilitate a successful plant invasion [22]. However, in *S. fredii* HH103, the picture that emerges from our results is quite different (Figure 3). 

First, despite of the fact that this bacterium also harbors functional *luxI*-type genes on its genome (*traI* and *sinI* that account for the synthesis of short- and long-chain AHLs, respectively), the concentration of AHLs in bacterial supernatants even at high population densities was not high enough to be detected by *A. tumefaciens* NT1 (pZRL4), which has been described as one of the most sensitive and versatile AHL biosensors [28]. In NGR234, the complete absence of AHLs triggers a mechanism that allows this bacterium to initiate the nodulation process in the absence of *nod*-gene inducing flavonoids: the copy number of the symbiotic plasmid is increased and consequently the relative expression of all symbiotic-related genes located in this plasmid is higher, which leads to NF production in a flavonoid-independent manner [62]. Could a similar mechanism be present in *S. fredii* HH103 due to the low production of AHLs? Results displayed in this manuscript support in some extend this hypothesis, since several copies of the HH013 pSym are present in this strain under the tested conditions. Besides, it has been recently described that HH103 produces low but detectable amounts of NF in the absence of inducing-flavonoids [67]. It remains to be elucidated whether this basal production of NF in *S. fredii* HH103 might be involved in symbiosis and/or biofilm formation, since these molecules are also part of the biofilm matrix of *S. meliloti* [68]. 

In addition, and according to the low AHLs production detected in HH103, both *traI* and *sinI* were barely expressed at stationary phase, suggesting that the typical positive feedback of the QS systems at high cellular density is not taking place in *S. fredii* HH103. In different RNAseq analyses performed by our group with HH103 and different mutants in symbiotic regulators (*nodD1*, *ttsI*, *nodD2*, *nolR*, *mucR*, and *syrM*), in the absence and presence of inducers (genistein or *Lotus japonicus* root exudates) we could not detect any changes in the expression rate of either *sinI* or *traI* [49,67,69,70]. Moreover, expression studies of the *sinI* gene carried out with the HH103 *sinI*::*lacZ*-Gm^R^ mutant revealed that the expression of this gene remains very low along the symbiotic interactions with soybean and *Lotus burttii* in either early and late steps of the nodulation process [70]. All these facts suggest that HH103 QS systems are inactive, most probably due to the mutations present in the *luxR*-type genes. However, although the cognate genes coding for the AHL-receptors of both *tra* and *sin* QS systems (*traR* and *expR*, respectively) are truncated, each one is divided into two ORFs, and the ones corresponding to the original C-term contain the complete transcription regulatory domain. Interestingly, deletion of the LuxR N-term domain in *Vibrio fisheri* results in a derivative protein that interacts with DNA and activates the transcription of target genes even in the absence of AHLs [60]. This finding has led to the hypothesis that in the native LuxR-type regulators, the N-term portion is reducing the DNA binding affinity of the C-term domain when AHLs are not present [71]. In *S. fredii* HH103, in contrast to the transcriptomic values obtained for *luxI*-type genes, ORFs that are coding for these C-term portions were highly expressed at high population levels. Therefore, these results point out that the TraR and ExpR C-term proteins could be expressed and controlling the QS-regulated phenotypes in an AHL-independent manner in HH103. The analysis of the transcriptomic rankings of putative ExpR-targets, such as *exo* genes, supports in some extend this hypothesis, since despite of the low AHLs concentration at high population densities, genes involved in the biosynthesis of EPS were highly expressed in contrast to chemotaxis and motility genes. Supporting this finding, the *sinI* and/or *traI* mutants of *S. fredii* HH103 showed similar mucoidy (indicative of EPS production) and swimming motility to those of the wild-type strain, which indicates that both phenotypes are occurring in an AHL-independent manner in this bacterium. In contrast, plasmid transfer genes did not display high transcriptional ranking, which could be indicating that the TraR C-term protein did not activate this set of genes under the tested conditions. In fact, we could not detect differences between the wild-type strain and its *traI* and/or *sinI* mutants in their abilities to transfer the symbiotic plasmid. Moreover, transcriptomic analysis indicated that the gene that codes for the anti-activator protein TraM is among the top 10% more transcribed genes of *S. fredii* HH103 under the tested conditions. This fact could explain why genes involved in plasmid transfer are not being activated by the TraR C-term protein at high population densities in the absence of AHLs, since TraR and TraM interact with each other through domains located at their respective C-term domains [65]. Interestingly, this could be a common phenomenon among *S. fredii* strains, since only 4 out of the 13 available *S. fredii* genomic sequences contain a complete *traR* gene. The control of plasmid transfer genes in HH103 might reside in other transcriptional regulators, among them some LuxR-orphan proteins of HH103 that might be sensing the AHLs produced by other bacteria.

In summary, we hypothesize that three phenotypes typically regulated by quorum sensing in sinorhizobia (swimming motility, EPS production and pSym conjugative transfer) have become AHL-independent in *S. fredii* HH103. Interestingly, flavonoids and NodD1 appear to have acquired the control of production of EPS, repressing its production [17], which might facilitate progression thought the infection threads and avoid plant defense responses at least in soybean, since lack of EPS production by *S. fredii* HH103 is not only non-detrimental but even beneficial for symbiosis with this host legume [72]. Remarkably, the same situation could be taking place for chemotaxis and motility phenotypes since, in contrast to that described for other *Sinorhizobium* strains, we have recently found that in the presence of inducing flavonoids HH103 activates surface motility in a NodD1-dependent manner [73]. Thus, in HH103, the production of EPS and the non-activation of surface motility might be linked to free-living lifestyle since it has been described that EPS provide protection against different stresses such as desiccation or the presence of antimicrobial compounds and have a role in biofilm formation [74,75], which is an opposite behavior to bacterial motility. Overall, in *S. fredii* HH103 the regulatory way mediated by NodD1 and flavonoids seems to have replace quorum sensing systems in the control of some physiological traits that are important for the transition between free-living and symbiotic lifestyles. Further efforts are needed to shed light to this hypothesis.

## Figures and Tables

**Table 1:** Bacterial strains and plasmid used in this study.

Strain or Plasmid	Relevant Properties	Source or Reference
***S. fredii***		
HH103	Wild-type strain, spontaneous Rif^R^	[27]
HH103 *traI*	HH103 *traI*::Ω, Spc^R^	This work
HH103 *sinI*	HH103 *sinI*::*lacZ*-Gm^R^, Gm^R^	This work
HH103 *traI sinI*	HH103 *traI*::Ω *sinI*::*lacZ*-Gm^R^, Spc^R^ Gm^R^	This work
*E. coli*		
DH5α	*fhuA2 lac(del)U169 phoA glnV44Φ80’ lacZ(del)M15 gyrA96 recA1 relA1 endA1 thi-1 hsdR17*, Nal^R^	Stratagene (USA)
***A. tumefaciens***		
NT1 (pZRL4)	*A. tumefaciens* devoid of pTiC58 and harboring pZRL4, which carries the fusion *traG*::*lacZ* and the *traR* gene	[28]
GMI9023 (pMUS248)	*A. tumefaciens* strain devoid of pTi and pAtC58 (C58 derivative), harboring plasmid pMUS248, Km^R^ and stable in rhizobia, which carries the fusion p*nodA*::*tet*Δp, Km^R^	[29]
**Plasmids**		
pRK2013	Helper plasmid, Km^R^	[30]
pGEM-T Easy	PCR cloning vector, Ap^R^	Promega (USA)
pK18mob	Cloning vector, suicide in rhizobia, Km^R^	[31]
pHP45Ω	Ap^R^ vector containing the Ω interposon, Ap^R^ Spc^R^	[32]
pAB2001	Ap^R^ vector containing the *lacZ*-Gm^R^ cassette, Ap^R^ Gm^R^	[33]
pMUS997	pGEM-T Easy::*traI*, Ap^R^	This work
pMUS1079	pGEM-T Easy::*sinI*, Ap^R^	This work
pMUS989	pK18mob::*traI*, Km^R^	This work
pMUS1083	pK18mob::*sinI*, Km^R^	This work
pMUS1006	pK18mob::*traI*::Ω, Km^R^ Spc^R^	This work
pMUS1087	pK18mob::*sinI*::*lacZ*-Gm^R^, Km^R^ Gm^R^	This work

**Table 2:** DNA oligonucleotide primers used in this study.

Name	Sequence	Usage
*traI* F	5′-CCAGAAGATTGGGATTGACA-3′	Amplification of the *traI* gene
*traI* R	5′-TGTCCGCCTATCGGAAGCTCA-3′	
*sinI* F	5′-TTTTCATGCGTCGATGCTCGA-3′	Amplification of the *sinI* gene
*sinI* R	5′-CCGTAGGTCG GAACAATGACA-3′	
*ligE*q_F	5′-AAGACCAAGCTGTCGCTC-3′	Chromosomal gene, *q*PCR assays
*ligE*q_R	5′-ATGTCGAAGCTGTCGCTG-3′	
*ftsZ1*q_F	5′-ATACGCTGATCGTCATCC-3′	Chromosomal gene, *q*PCR assays
*ftsZ1*q_R	5′-GCCTTCCTTGACCATGAG-3′	
*flgJ*q_F	5′-TGCTGAATTCCTCGGAAG-3′	Chromosomal gene, *q*PCR assays
*flgJ*q_R	5′-CAGCATCGACTTGACGAA-3′	
*nolR*q_F	5′-CCAAAACGCCTGCTCATT-3′	Chromosomal gene, *q*PCR assays
*nolR*q_R	5′-ATTCTGGGCACGCAACTT-3′	
*nodA*q_F	5′-ACGTCATGTATCCGGTGCTGCA-3′	pSym gene, *q*PCR assays
*nodA*q_R	5′-CGTTGGCGGCAGGTTGAGA-3′	
*syrM*q_F	5′-GTTCAATGACGATCTCTTGGT-3′	pSym gene, *q*PCR assays
*syrM*q_R	5′-ATTGCCATAGTTACCTTCGAC-3′	
*d373*q_F	5′-TCGACGATTCAATAAGGGTG-3′	pSym gene, *q*PCR assays
*d373*q_R	5′-CATATCCTCTCCGCAATAGC-3′	
*d161*q_F	5′-AGAATGTCGCATACCTCTTAG-3′	pSym gene, *q*PCR assays
*d161*q_R	5′-GTGAAGGCTGTTATCCCATC-3′	
q*nodD1*-F	5′-GCGAGCACGGACTGCGAA-3′	pSym gene, confirmation of conjugation transfer of this plasmid to GMI9023 (pMUS248)
q*nodD1*-R	5′-CGGGAAAAATGGGTTGCGGA-3′	

**Table 3:** AHLs identified by HPLC-MS/MS in supernatants of *S. fredii* strains (+: detected, −: not detected).

Standars	Strain			
	HH103	*traI*	*sinI*	*traI*/*sinI*
C6-HSL	−	−	−	−
3-oxo-C6-HSL	−	−	−	−
C8-HSL	+	−	+	−
3-oxo-C8-HSL	+	−	+	−
C10-HSL	−	−	−	−
3-oxo-C10-HSL	−	−	−	−
C12-HSL	+	+	−	−
3-oxo-C12-HSL	−	−	−	−
3-OH-C12-HSL	−	−	−	−
C14-HSL	+	+	−	−
3-oxo-C14-HSL	+	+	−	−
3-OH-C14-HSL	−	+	−	−

**Table 4:** Number of transcripts assigned to putative quorum sensing (QS)-regulated genes of *S. fredii* HH103 and transcriptional ranking in yeast extract mannitol (YM) medium at stationary phase in the absence of inducing flavonoids.

Gene ID	Gene Name	Number of Transcripts	Relative Position (among 7014 Total Number of ORFs)	Transcriptomic Ranking
**Quorum sensing genes**				
psfHH103d_478	*traI*	54	5662	19.3
SFHH103_01572	*sinI*	82	4910	30.0
psfHH103d_461	*traM*	2208	500	92.9
psfHH103d_462	*traR end*	541	1629	76.8
psfHH103d_463	*traR start*	1730	638	90.9
SFHH103_1571	*sinR*	512	1704	75.7
SFHH103_03306	*expR end*	450	1863	73.4
SFHH103_03432	*expR start*	350	2207	68.5
**Nodulation genes**				
psfHH103d_386	*nodD1*	1579	696	90.1
psfHH103d_126	*nodA*	75	5090	27.4
psfHH103d_127	*nodB*	156	3611	48.5
psfHH103d_128	*nodC*	316	2363	66.3
psfHH103d_129	*nodI*	86	4819	31.3
psfHH103d_130	*nodJ*	116	4199	40.1
psfHH103d_131	*nolO’*	376	2101	70.0
psfHH103d_132	*noeI*	186	3281	53.2
psfHH103d_381	*nodZ*	1022	979	86.0
psfHH103d_380	*noeL*	571	1562	77.7
psfHH103d_339	*nolU*	189	3243	53.8
**Average**		**425**	**2904**	**58.6**
**Chemotaxis and motility genes**				
SFHH103_00293	*mcpE*	107	4367	37.7
SFHH103_00294	*cheX*	23	6642	5.3
SFHH103_00295	*cheY1*	21	6700	4.5
SFHH103_00296	*cheA*	128	3200	54.4
SFHH103_00297	*cheW1*	9	6939	1.1
SFHH103_00298	*cheR*	30	6436	8.2
SFHH103_00299	*cheB*	46	5931	15.4
SFHH103_00300	*cheY2*	33	6345	9.5
SFHH103_00301	*cheD*	30	6437	8.2
SFHH103_00303	*fliF*	108	4341	38.1
SFHH103_00304	*visN*	660	1410	79.9
SFHH103_00305	*visR*	489	1757	75.0
SFHH103_00307	*flhB*	142	3775	46.2
SFHH103_00308	*fliG*	45	5958	15.1
SFHH103_00309	*fliN*	44	5993	14.6
SFHH103_00310	*fliM*	37	6215	11.4
SFHH103_00311	*motA*	37	6225	11.2
SFHH103_00313	*flgF*	29	6454	8.0
SFHH103_00314	*fliI*	42	6058	13.6
SFHH103_00316	*flgB*	51	5757	17.9
SFHH103_00317	*flgC*	16	6819	2.8
SFHH103_00318	*fliE*	21	6686	4.7
SFHH103_00319	*flgG*	29	6469	7.8
SFHH103_00320	*flgA*	29	6470	7.8
SFHH103_00321	*flgI*	27	6534	6.8
SFHH103_00323	*flgH*	48	5857	16.5
SFHH103_00324	*fliL*	44	5991	14.6
SFHH103_00325	*fliP*	135	3886	44.6
SFHH103_00326	*flaC*	493	1746	75.1
SFHH103_00327	*flaB*	592	1516	78.4
SFHH103_00328	*flaA*	193	3192	54.5
SFHH103_00329	*flaD*	140	3810	45.7
SFHH103_00331	*motB*	82	4918	29.9
SFHH103_00332	*motC*	59	5549	20.9
SFHH103_00333	*motD*	93	4647	33.7
SFHH103_00336	*flgE*	84	4865	30.6
SFHH103_00337	*flgK*	66	5315	24.2
SFHH103_00338	*flgL*	103	4446	36.6
SFHH103_00339	*flaF*	61	5498	21.6
SFHH103_00340	*flbT*	62	5444	22.4
SFHH103_00341	*flgD*	66	5338	23.9
SFHH103_00342	*fliQ*	135	3890	44.5
SFHH103_00343	*flhA*	119	4144	40.9
SFHH103_00344	*fliR*	75	5092	27.4
SFHH103_00346	*flgJ*	27	6541	6.7
**Average**		**109**	**5147**	**26.6**
**Exopolysaccharydes genes**				
SFHH103_01240	*exoR*	27591	27	99.6
SFHH103_02875	*exoN*	2678	377	94.6
SFHH103_03541	*exoS*	777	1237	82.4
SFHH103_03846	*exoD*	1785	623	91.1
SFHH103_05372	*exoP*	7993	110	98.4
SFHH103_05373	*exoN*	2916	422	94.0
SFHH103_05374	*exoO*	1191	881	87.4
SFHH103_05375	*exoM*	2698	420	94.0
SFHH103_05376	*exoA*	4029	270	96.2
SFHH103_05377	*exoL*	7280	124	98.2
SFHH103_05378	*exoK*	15966	52	99.3
SFHH103_05380	*exoI*	278	2537	63.8
SFHH103_05382	*exoU*	2212	498	92.9
SFHH103_05383	*exoX*	787	1222	82.6
SFHH103_05384	*exoY2*	33321	23	99.7
SFHH103_05386	*exoF1*	5959	160	97.7
SFHH103_05387	*exoQ*	1783	625	91.1
SFHH103_05388	*exoZ*	981	1020	85.5
SFHH103_05389	*exoB*	7200	126	98.2
SFHH103_05659	*exoF2*	66	5332	24.0
SFHH103_05660	*exoY1*	123	4088	41.7
SFHH103_05850	*exoF3*	519	1686	76.0
**Average**		**5824**	**994**	**85.8**
**Plasmid transfer genes**				
psfHH103d_56	*traG*	75	5082	27.5
psfHH103d_57	*traD*	16	6820	2.8
psfHH103d_57_5	*traC*	20	6721	4.2
psfHH103d_58	*traA*	260	2638	62.4
psfHH103d_465	*trbI*	87	4801	31.6
psfHH103d_466	*trbH*	25	6570	6.3
psfHH103d_467	*trbG*	23	6643	5.3
psfHH103d_468	*trbF*	24	6612	5.7
psfHH103d_469	*trbL*	65	5364	23.5
psfHH103d_470	*trbK*	17	6777	3.4
psfHH103d_471	*trbJ*	49	5820	17.0
psfHH103d_472	*trbE*	68	5265	24.9
psfHH103d_475	*trbD*	42	6055	13.7
psfHH103d_476	*trbC*	35	6292	10.3
psfHH103d_477	*trbB*	60	5531	21.1
SFHH103_06246	*traA*	244	2740	60.9
SFHH103_06247	*traD*	48	5878	16.2
SFHH103_06248	*traG*	116	4200	40.1
SFHH103_03975	*traG*	176	3396	51.6
SFHH103_03977	*traA*	1219	861	87.7
SFHH103_03995	*traG*	362	2158	69.2
SFHH103_03999	*traA*	1443	746	89.4
**Average**		**203**	**4862**	**30.7**

## Supplementary Materials

The following are available online at https://www.mdpi.com/2076-2607/8/1/68/s1. Figure S1: Well diffusion assays of bacterial supernatants obtained from *Sinorhizobium fredii* HH103; Figure S2: Growth curves of the different *Sinorhizobium fredii* HH103 strains; Figure S3: KPS, LPS and EPS production by *Sinorhizobium fredii* HH103 strains; Figure S4: Biofilm formation and swimming motility of the different *Sinorhizobium fredii* HH103 strains. Table S1: Plant responses to inoculation of *Glycine max* or *Glycyrrhiza uralensis* with *Sinorhizobium fredii* HH103 strains. Table S2: Report of a BLASTp search of the *Sinorhizobium fredii* NGR234 TraR protein against all *Sinorhizobium fredii* genomes available on the JGI- IMG public repository. Table S3: Report of a BLASTp search of the *Sinorhizobium fredii* NGR234 SinR protein against all *Sinorhizobium fredii* genomes available on the JGI- IMG public repository. Table S4: Report of a BLASTp search of the *Sinorhizobium fredii* NGR234 ExpR protein against all *Sinorhizobium fredii* genomes available on the JGI- IMG public repository.

## References

[1] Gage D.J. (2004). Infection and invasion of roots by symbiotic, nitrogen-fixing rhizobia during nodulation of temperate legumes. Microbiol. Mol. Biol. Rev..

[2] Jones K.M., Kobayashi H., Davies B.W., Taga M.E., Walker G.C. (2007). How rhizobial symbionts invade plants: The *Sinorhizobium-Medicago* model. Nat. Rev. Microbiol..

[3] Deakin W.J., Broughton W.J. (2009). Symbiotic use of pathogenic strategies, rhizobial protein secretion systems. Nat. Rev. Microbiol..

[4] Spaink H.P. (2000). Root nodulation and infection factors produced by rhizobial bacteria. Annu. Rev. Microbiol..

[5] Mierziak J., Kostyn K., Kulma A. (2014). Flavonoids as important molecules of plant interactions with the environment. Molecules.

[6] Nelson M.S., Sadowsky M.J. (2015). Secretion systems and signal exchange between nitrogen-fixing rhizobia and legumes. Front. Plant Sci..

[7] Kondorosi E., Gyuris J., Schmidt J., John M., Duda E., Hoffmann B., Schell J., Kondorosi A. (1989). Positive and negative control of *nod* gene expression in *Rhizobium meliloti* is required for optimal nodulation. EMBO J..

[8] Oldroyd G.E. (2013). Speak, friend, and enter: Signaling systems that promote beneficial symbiotic associations in plants. Nat. Rev. Microbiol..

[9] Broughton W.J., Perret X. (1999). Genealogy of legume- *Rhizobium* symbioses. Curr. Opin. Plant Biol..

[10] Krysciak D., Orbegoso M.R., Schmeisser C., Streit W.R., de Bruijn F.J. (2015). Molecular keys to broad host range in *Sinorhizobium fredii* NGR234, USDA257, and HH103. Biological Nitrogen Fixation.

[11] Pueppke S.G., Broughton W.J. (1999). *Rhizobium* sp. Strain NGR234 and *R. fredii* USDA257 share exceptionally broad, nested host ranges. Mol. Plant Microbe Interact..

[12] Schmeisser C., Liesegang H., Krysciak D., Bakkou N., Le Quéré A., Wollherr A., Heinemeyer I., Morgenstern B., Pommerening-Röser A., Flores M. (2009). *Rhizobium* sp. strain NGR234 possesses a remarkable number of secretion systems. Appl. Environ. Microbiol..

[13] Schuldes J., Rodriguez-Orbegoso M., Schmeisser C., Krishnan H.B., Daniel R., Streit W.R. (2012). Complete genome sequence of the broad-host-range strain *Sinorhizobium fredii* USDA257. J. Bacteriol..

[14] Vinardell J.M., Acosta-Jurado S., Zehner S., Göttfert M., Becker A., Baena I., Blom J., Crespo-Rivas J.C., Goesmann A., Jaenicke S. (2015). The *Sinorhizobium fredii* HH103 genome: A comparative analysis with *S. fredii* strains differing in their symbiotic behavior with soybean. Mol. Plant Microbe Interact..

[15] López-Baena F.J., Vinardell J.M., Pérez-Montano F., Crespo-Rivas J.C., Bellogín R.A., Espuny M.R., Ollero F.J. (2008). Regulation and symbiotic significance of nodulation outer proteins secretion in *Sinorhizobium fredii* HH103. Microbiology.

[16] Pérez-Montaño F., Guasch-Vidal B., González-Barroso S., López-Baena F.J., Cubo T., Ollero F.J., Gil-Serrano A.M., Rodríguez-Carvajal M.Á., Bellogín R.A., Espuny M.R. (2011). Nodulation-gene-inducing flavonoids increase overall production of autoinducers and expression of N-acyl homoserine lactone synthesis genes in rhizobia. Res. Microbiol..

[17] Acosta-Jurado S., Navarro-Gómez P., Murdoch P.S., Crespo-Rivas J.C., Jie S., Cuesta-Berrio L., Ruiz-Sainz J.E., Rodríguez-Carvajal M.Á., Vinardell J.M. (2016). Exopolysaccharide Production by *Sinorhizobium fredii* HH103 Is Repressed by Genistein in a NodD1-Dependent Manner. PLoS ONE.

[18] Downie J.A. (2010). The roles of extracellular proteins, polysaccharides and signals in the interactions of rhizobia with legume roots. FEMS Microbiol. Rev..

[19] Wisniewski-Dyé F., Downie J.A. (2002). Quorum-sensing in *Rhizobium*. Antonie Leeuwenhoek.

[20] González J.E., Marketon M.M. (2003). Quorum sensing in nitrogen-fixing rhizobia. Microbiol. Mol. Biol. Rev..

[21] Sánchez-Contreras M., Bauer W.D., Gao M., Robinson J.B., Allan Downie J. (2007). Quorum-sensing regulation in rhizobia and its role in symbiotic interactions with legumes. Philos. Trans. R. Soc. Lond B Biol. Sci..

[22] Calatrava-Morales N., McIntosh M., Soto M.J. (2018). Regulation Mediated by *N*-acyl homoserine lactone quorum sensing signals in the *Rhizobium*-legume sSymbiosis. Genes.

[23] Beringer J.E. (1974). R factor transfer in *Rhizobium leguminosarum*. J. Gen. Microbiol..

[24] Vincent J.M. (1970). The modified Fåhraeus slide technique. A Manual for the Practical Study of Root Nodule Bacteria.

[25] Bertani G. (1951). Studies on lysogenesis. I. The mode of phage liberation by lysogenic *Escherichia coli*. J. Bacteriol..

[26] Lamrabet Y., Bellogín R.A., Cubo T., Espuny M.R., Gil-Serrano A., Krishnan H.B., Megias M., Ollero F.J., Pueppke S.G., Ruiz-Sainz J.E. (1999). Mutation in GDP fucose synthesis genes of *Sinorhizobium fredii* alters Nod factors and significantly decreases competitiveness to nodulate soybeans. Mol. Plant Microbe Interact..

[27] Madinabeitia N., Bellogín R.A., Buendía-Clavería A.M., Camacho M., Cubo T., Espuny M.R., Gil-Serrano A.M., Lyra M.C., Moussaid A., Ollero F.J. (2002). *Sinorhizobium fredii* HH103 has a truncated *nolO* gene due to a -1 frameshift mutation that is conserved among other geographically distant *S. fredii* strains. Mol. Plant Microbe Interact..

[28] Cha C., Gao P., Chen Y.C., Shaw P.D., Farrand S.K. (1998). Production of acyl-homoserine lactone quorum-sensing signals by gram-negative plant associated bacteria. Mol. Plant Microbe Interact..

[29] Vinardell J.M., López-Baena F.J., Hidalgo A., Ollero F.J., Bellogín R., Espuny M.R., Temprano F., Romero F., Krishnan H.B., Pueppke S.G. (2004). The effect of FITA mutations on the symbiotic properties of *Sinorhizobium fredii* varies in a chromosomal-background-dependent manner. Arch. Microbiol..

[30] Figurski D.H., Helinski D.R. (1979). Replication of an origin-containing derivative of plasmid RK2 dependent on a plasmid function provided in trans. Proc. Natl. Acad. Sci. USA.

[31] Schäfer A., Tauch A., Jager W., Kalinowski J., Thierbach G., Pühler A. (1995). Small mobilizable multi-purpose cloning vectors derived from the *Escherichia coli* plasmids pK18 and pK19: Selection of defined deletions in the chromosome of *Corynebacterium glutamicum*. Gene.

[32] Prentki P., Krisch H.M. (1984). In vitro insertional mutagenesis with a selectable DNA fragment. Gene.

[33] Becker A., Schmidt M., Jäger W., Pühler A. (1995). New gentamicin-resistance and *lacZ* promoter-probe cassettes suitable for insertion mutagenesis and generation of transcriptional fusions. Gene.

[34] Simon R. (1984). High frequency mobilization of gram-negative bacterial replicons by the in vitro constructed TnS-Mob transposon. Mol. Gen. Genet..

[35] Sambrook J., Fritsch E.F., Maniatis T. (1989). Molecular Cloning. A Laboratory Manual.

[36] López-Baena F.J., Monreal J.A., Pérez-Montaño F., Guasch-Vidal B., Bellogín R.A., Vinardell J.M., Ollero F.J. (2009). The absence of Nops secretion in *Sinorhizobium* fredii HH103 increases GmPR1 expression in Williams soybean. Mol. Plant Microbe Interact..

[37] McClean K.H., Winson M.K., Fish L., Taylor A., Chhabra S.R., Camara M., Daykin M., Lamb J.H., Swift S., Bycroft B.W. (1997). Quorum sensing and *Chromobacterium violaceum*: Exploitation of violacein production and inhibition for the detection of N-acylhomoserine lactones. Microbiology.

[38] Morin D., Grasland B., Vallee-Rehel K., Dufau C., Haras D. (2003). On-line high-performance liquid chromatography-mass spectrometric detection and quantification of N-acylhomoserinelactones, quorum sensing signal molecules, in the presence of biological matrices. J. Chromatogr..

[39] Cataldi T.R.I., Bianco G., Abate S., Mattia D. (2009). Analysis of S-adenosylmethionine and related sulfur metabolites in bacterial isolates of *Pseudomonas aeruginosa* (BAA-47) by liquid chromatography/electrospray ionization coupled to a hybrid linear quadrupole ion trap and fourier transform ion cyclotron resonance mass spectrometry. Rapid Commun. Mass Spectrom..

[40] Gould T.A., Herman J., Krank J., Murphy R.C., Churchill M.E. (2006). Specificity of acyl-homoserine lactone synthases examined by mass spectrometry. J. Bacteriol..

[41] Kumari A., Pasini P., Daunert S. (2008). Detection of bacterial quorum sensing N-acyl homoserine lactones in clinical samples. Anal. Bioanal. Chem..

[42] Makemson J., Eberhard A., Mathee K. (2006). Simple electrospray mass spectrometry detection of acylhomoserine lactones. Luminescence.

[43] Ortori C.A., Atkinson S., Chhabra S.R., Cámara M., Williams P., Barrett D.A. (2007). Comprehensive profiling of N-acylhomoserine lactones produced by *Yersinia pseudotuberculosis* using liquid chromatography coupled to hybrid quadrupole-linear ion trap mass spectrometry. Anal. Bioanal. Chem..

[44] Buendía-Clavería A.M., Moussaid A., Ollero F.J., Vinardell J.M., Torres A., Moreno J., Gil-Serrano A.M., Rodríguez-Carvajal M.A., Tejero-Mateo P., Peart J.L. (2003). A *purL* mutant of *Sinorhizobium fredii* HH103 is symbiotically defective and altered in its lipopolysaccharide. Microbiology.

[45] Hidalgo A., Margaret I., Crespo-Rivas J.C., Parada M., Murdoch P.S., López A., Buendía-Clavería A.M., Moreno J., Albareda M., Gil-Serrano A.M. (2010). The *rkpU* gene of *Sinorhizobium fredii* HH103 is required for bacterial K-antigen polysaccharide production and for efficient nodulation with soybean but not with cowpea. Microbiology.

[46] Sourjik V., Schmitt R. (1996). Different roles of CheY1 and CheY2 in the chemotaxis of *Rhizobium meliloti*. Mol. Microbiol..

[47] O’Toole G.A., Kolter R. (1998). Initiation of biofilm formation in *Pseudomonas fluorescens* WCS365 proceeds via multiple, convergent signalling pathways: A genetic analysis. Mol. Microbiol..

[48] Mueller K., González J.E. (2011). Complex regulation of symbiotic functions is coordinated by MucR and quorum sensing in *Sinorhizobium meliloti*. J. Bacteriol..

[49] Pérez-Montaño F., Jiménez-Guerrero I., Acosta-Jurado S., Navarro-Gómez P., Ollero F.J., Ruiz-Sainz J.E., López-Baena F.J., Vinardell J.M. (2016). A transcriptomic analysis of the effect of flavonoids on *Sinorhizobium fredii* HH103 reveals novel rhizobial genes putatively involved in symbiosis. Sci. Rep..

[50] Yang R., Santos-Garcia D., Pérez-Montaño F., da Silva G.M., Zhao M., Jiménez-Guerrero I., Rosenberg T., Chen G., Plaschkes I., Morin S. (2019). Complete Assembly of the Genome of an *Acidovorax citrulli* Strain Reveals a Naturally Occurring Plasmid in This Species. Front. Microbiol..

[51] Hoang H.H., Becker A., González J.E. (2004). The LuxR homolog ExpR, in combination with the Sin quorum sensing system, plays a central role in *Sinorhizobium meliloti* gene expression. J. Bacteriol..

[52] López-Baena F.J., Ruiz-Sainz J.E., Rodríguez-Carvajal M.A., Vinardell J.M. (2016). Bacterial Molecular Signals in the *Sinorhizobium fredii*-Soybean Symbiosis. Int. J. Mol. Sci..

[53] Pérez-Montaño F., Jiménez-Guerrero I., Del Cerro P., Baena-Ropero I., López-Baena F.J., Ollero F.J., Bellogín R., Lloret J., Espuny R. (2014). The Symbiotic Biofilm of *Sinorhizobium fredii* SMH12, Necessary for Successful Colonization and Symbiosis of *Glycine max* cv Osumi, is Regulated by Quorum Sensing Systems and Inducing Flavonoids via NodD1. PLoS ONE.

[54] Marketon M.M., Gronquist M.R., Eberhard A., González J.E. (2002). Characterization of the *Sinorhizobium meliloti sinR/sinI* locus and the production of novel N-acyl homoserine lactones. J. Bacteriol..

[55] Marketon M.M., González J.E. (2002). Identification of two quorum-sensing systems in *Sinorhizobium meliloti*. J. Bacteriol..

[56] Charoenpanich P., Meyer S., Becker A., McIntosh M. (2013). Temporal expression program of quorum sensing-based transcription regulation in *Sinorhizobium meliloti*. J. Bacteriol..

[57] Gao M., Chen H., Eberhard A., Gronquist M.R., Robinson J.B., Rolfe B.G., Bauer W.D. (2005). sinI- and expR-dependent quorum sensing in *Sinorhizobium meliloti*. J. Bacteriol..

[58] Gurich N., González J.E. (2009). Role of quorum sensing in *Sinorhizobium meliloti*-alfalfa symbiosis. J. Bacteriol..

[59] Nasser W., Reverchon S. (2007). New insights into the regulatory mechanisms of the LuxR family of quorum sensing regulators. Anal. Bioanal. Chem..

[60] Choi S.H., Greenberg E.P. (1991). The C-terminal region of the *Vibrio fischeri* LuxR protein contains an inducer-independent lux gene activating domain. Proc. Natl. Acad. Sci. USA.

[61] Fuqua W.C., Winans S.C. (1994). A LuxR-LuxI type regulatory system activates *Agrobacterium* Ti plasmid conjugal transfer in the presence of a plant tumor metabolite. J. Bact..

[62] Grote J., Krysciak D., Petersen K., Gullert S., Schmeisser C., Forstner K.U., Krishnan H.B., Schwalbe H., Kubatova N., Streit W.R. (2016). The absence of the N-acyl-homoserine-lactone autoinducer synthase genes *traI* and *ngrI* increases the copy number of the symbiotic plasmid in *Sinorhizobium fredii* NGR234. Front. Microbiol..

[63] He X., Chang W., Pierce D.L., Seib L.O., Wagner J., Fuqua C. (2003). Quorum sensing in *Rhizobium* sp. Strain NGR234 regulates conjugal transfer (*tra*) gene expression and influences growth rate. J. Bacteriol..

[64] Krysciak D., Grote J., Rodriguez Orbegoso M., Utpatel C., Forstner K.U., Li L., Schmeisser C., Krishnan H.B., Streit W.R. (2014). RNA sequencing analysis of the broad-host-range strain *Sinorhizobium fredii* NGR234 identifies a large set of genes linked to quorum sensing-dependent regulation in the background of a *traI* and *ngrI* deletion mutant. Appl. Environ. Microbiol..

[65] Hwang I., Smyth A.J., Luo Z.Q., Farrand S.K. (1999). Modulating quorum sensing by antiactivation: TraM interacts with TraR to inhibit activation of Ti plasmid conjugal transfer genes. Mol. Microbiol..

[66] McIntosh M., Meyer S., Becker A. (2009). Novel *Sinorhizobium meliloti* quorum sensing positive and negative regulatory feedback mechanisms respond to phosphate availability. Mol. Microbiol..

[67] Acosta-Jurado S., Rodríguez-Navarro D.N., Kawaharada Y., Rodríguez-Carvajal M.A., Gil-Serrano A., Soria-Díaz M.E., Pérez-Montaño F., Fernández-Perea J., Niu Y., Alias-Villegas C. (2019). *Sinorhizobium fredii* HH103 *nolR* and *nodD2* mutants gain capacity for infection thread invasion of *Lotus japonicus* Gifu and *Lotus burttii*. Environ. Microbiol..

[68] Fujishige N.A., Lum M.R., De Hoff P.L., Whitelegge J.P., Faull K.F., Hirsch A.M. (2008). *Rhizobium* common nod genes are required for biofilm formation. Mol. Microbiol..

[69] Acosta-Jurado S., Alias-Villegas C., Navarro-Gómez P., Zehner S., Murdoch P.D.S., Rodríguez-Carvajal M.A., Soto M.J., Ollero F.J., Ruiz-Sainz J.E., Göttfert M. (2016). The *Sinorhizobium fredii* HH103 MucR1 global regulator is connected with the nod regulon and is required for efficient symbiosis with *Lotus burttii* and *Glycine max* cv. Williams. Mol. Plant Microbe Interact..

[70] Acosta-Jurado S., Alias-Villegas C., Navarro-Gómez P., Almozara A., Rodríguez-Carvajal M.A., Medina C., Vinardell J.M. (2019). *Sinorhizobium fredii* HH103 *syrM* inactivation affects the expression of a large number of genes, impairs nodulation with soybean, and extends the host-range to the *Lotus japonicus*. Environ. Microbiol..

[71] Luo Z.Q., Farrand S.K. (1999). Signal-dependent DNA binding and functional domains of the quorum-sensing activator TraR as identified by repressor activity. Proc. Natl. Acad. Sci. USA.

[72] Parada M., Vinardell J.M., Ollero F.J., Hidalgo A., Gutiérrez R., Buendía-Clavería A.M., Lei W., Margaret I., López-Baena F.J., Gil-Serrano A.M. (2006). *Sinorhizobium fredii* HH103 mutants affected in capsular polysaccharide (KPS) are impaired for nodulation with soybean and *Cajanus cajan*. Mol. Plant Microbe Interact..

[73] Alías-Villegas C., Navarro-Gómez P., Almorzara A., Soto M.J., Vinardell J.M., Acosta-Jurado S. (2019). Surface motility in *Sinorhizobium fredii* HH103 Is activated by Genistein in a NodD1-Dependent Manner.

[74] Fraysse N., Couderc F., Poinsot V. (2003). Surface polysaccharide involvement in establishing the *Rhizobium* legume symbiosis. Eur. J. Biochem..

[75] Janczarek M. (2011). Environmental signals and regulatory pathways that influence exopolysaccharide production in rhizobia. Int. J. Mol. Sci..

