# Supplementary material for: Deciphering the Symbiotic Significance of Quorum Sensing Systems of Sinorhizobium fredii HH103
Source: Microorganisms. 2020 Jan 2;8(1):68. doi: 10.3390/microorganisms8010068 (PMC7022240; doi:10.3390/microorganisms8010068)
Supplement: Supplementary file 1 [file microorganisms-08-00068-s001.zip › Table S1.docx]

**Table S1.** Nodule numbers, fresh mass of nodules and plant-top dry mass of *G. max* with *S. fredii* strain HH103 and derivatives.

|  | ***Glycine max*** | | | ***Glycyrrhiza uralensis***^b^ | |
| --- | --- | --- | --- | --- | --- |
|  | Nodule number | Fresh mass of nodules (mg) | Plant top dry mass (g) | Number of plants nodulated/inoculated | Plant development |
| non-inoculated | 0.0±0.0 | 0.0±0.0 | 0.29±0.02b | 0/0 |  |
| HH103 | 37.0±2.5a | 929.50±37.72a | 1.14±0.06a | 17/19 (89.5%) | 3/19 (15.8%) |
| *traI* | 39.6±3.2a | 892.75±48.94a | 1.07±0.08a | 11/14 (78.6%) | 2/14 (14.3%) |
| *sinI* | 40.5±2.8a | 961.50±49.95a | 1.09±0.05a | 15/17 (88.2%) | 1/17 (5.9%) |
| *traI/sinI* | 45.3±5.3a | 859.83±61.47a | 0.99±0.07a | 19/21 (90.5%) | 1/21 (4.8%) |

^a^Data represent means ± SEM (standard error of the mean) of three experiments with six replicates for each treatment. Data in the same column with the same letter were not significantly different (One-Way ANOVA, α = 5%). Data for *G. uralensis* plants were made 8 weeks after inoculation. Each jar contained three plants.

^b^ For each inoculant each fraction represents the number of plants out of the total number of plants inoculated in which their plant-top dry weight (PTDW) was at least three-fold-higher than the PTDW mean value (45.95 mg ± 18.35) of the uninoculated control. Number in brackets refers to % values of “Number of plants nodulated/Number of plants inoculated” or to % values of the number of inoculated plants that reached a PTDW that was at least three-fold higher than that of the uninoculated control.
