# Supplementary figures and images for: Deciphering the Symbiotic Significance of Quorum Sensing Systems of Sinorhizobium fredii HH103
Source: Microorganisms. 2020 Jan 2;8(1):68. doi: 10.3390/microorganisms8010068 (PMC7022240; doi:10.3390/microorganisms8010068)

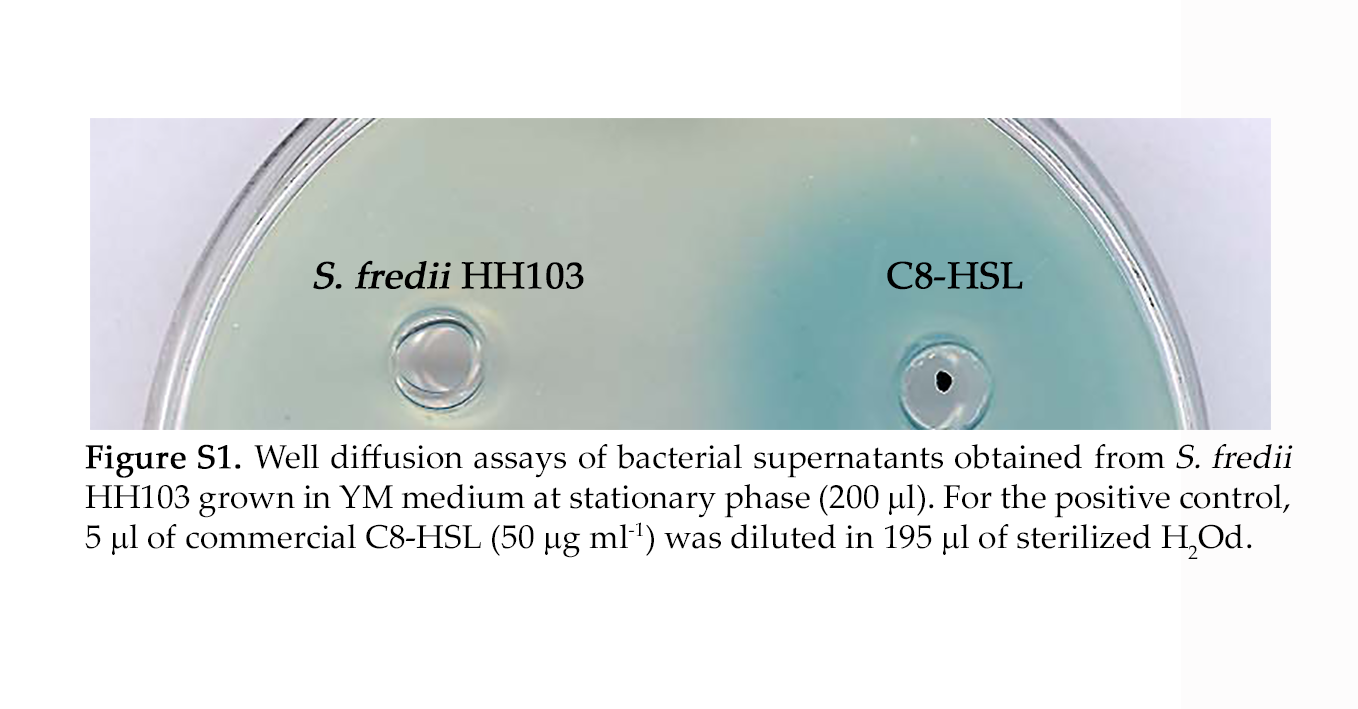

Supplement: Supplementary file 1 [file microorganisms-08-00068-s001.zip › Figure S1 combinado.tif]

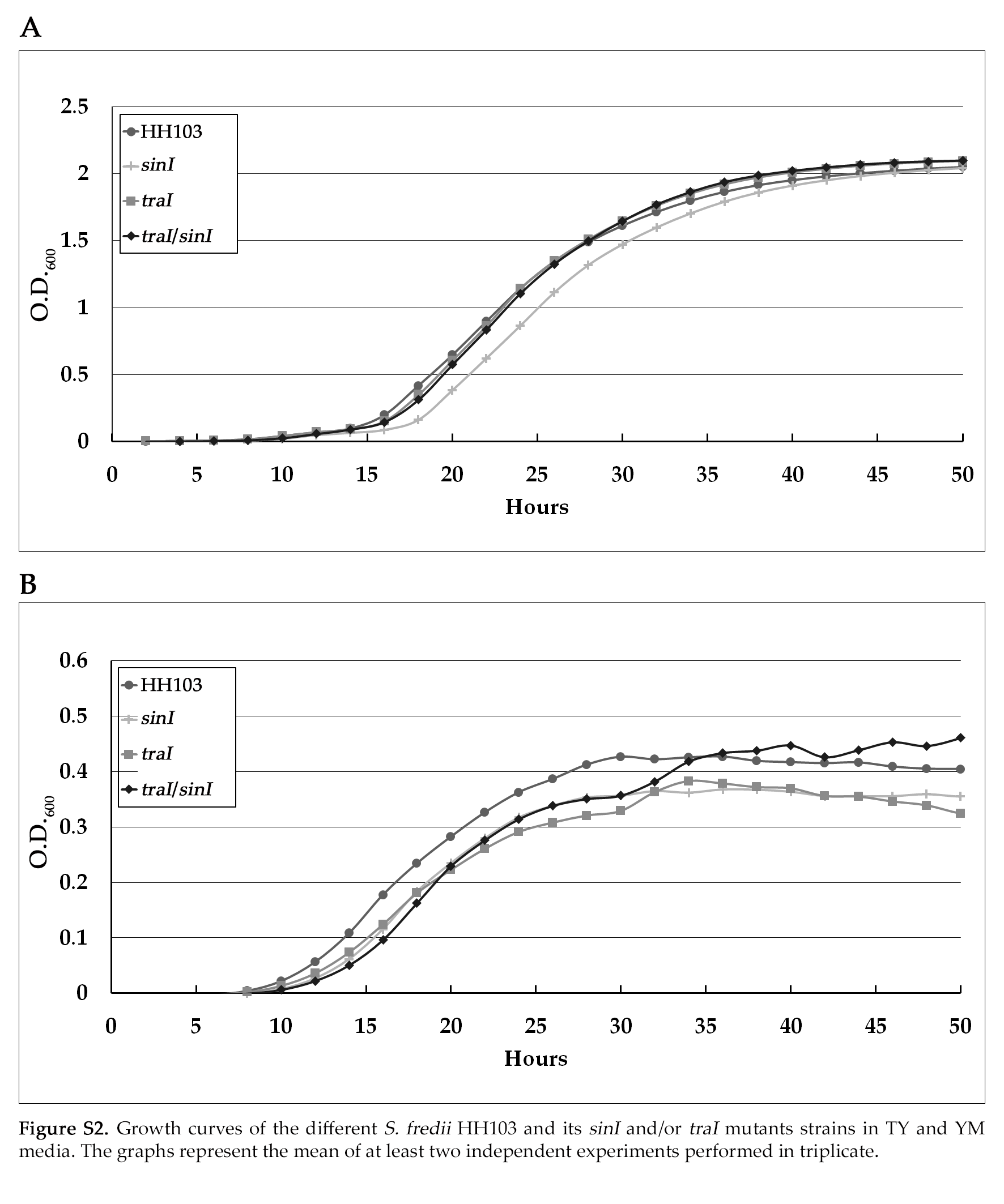

Supplement: Supplementary file 1 [file microorganisms-08-00068-s001.zip › Figure S2 combinado.tif]

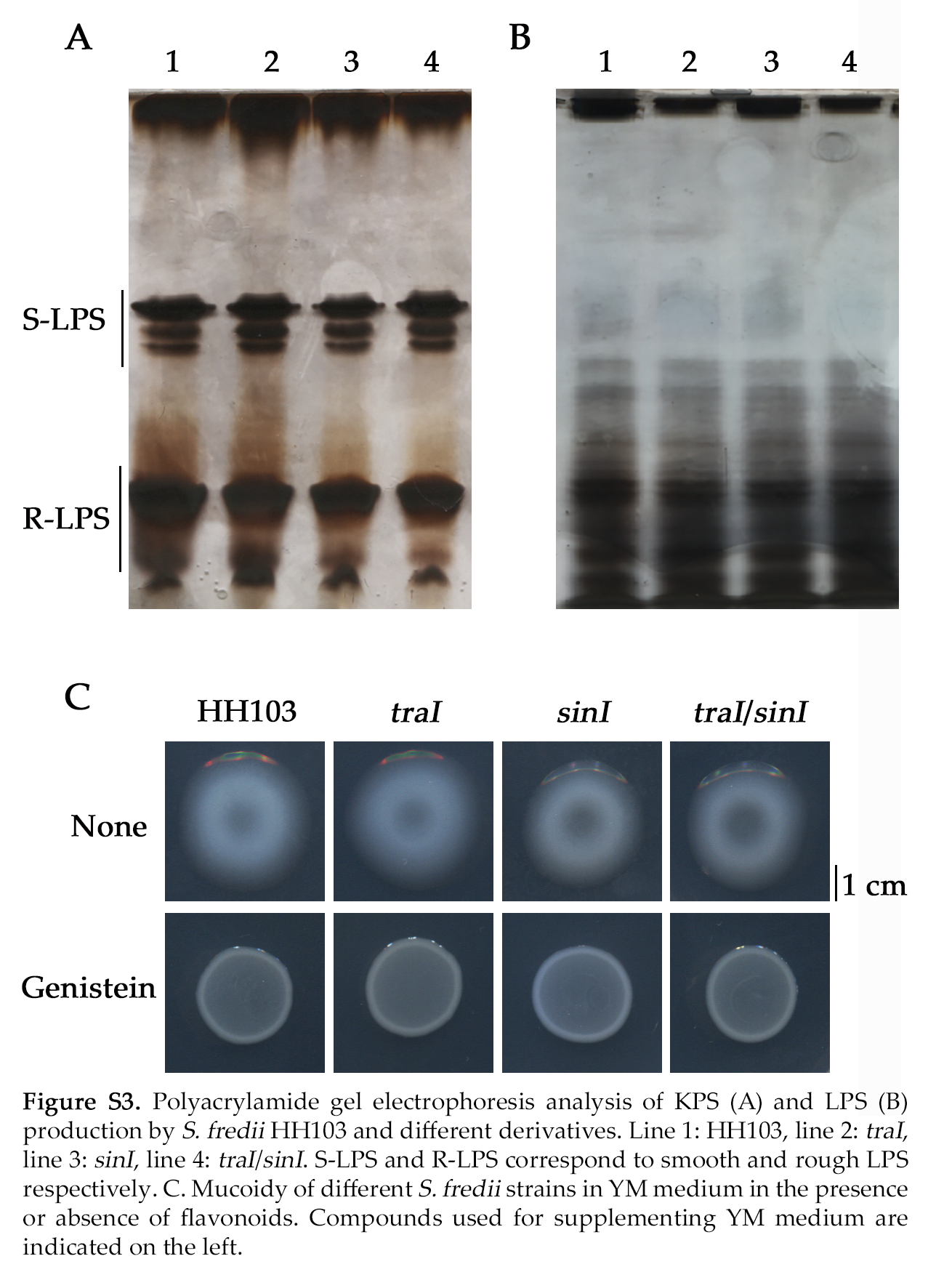

Supplement: Supplementary file 1 [file microorganisms-08-00068-s001.zip › Figure S3 combinado.tif]

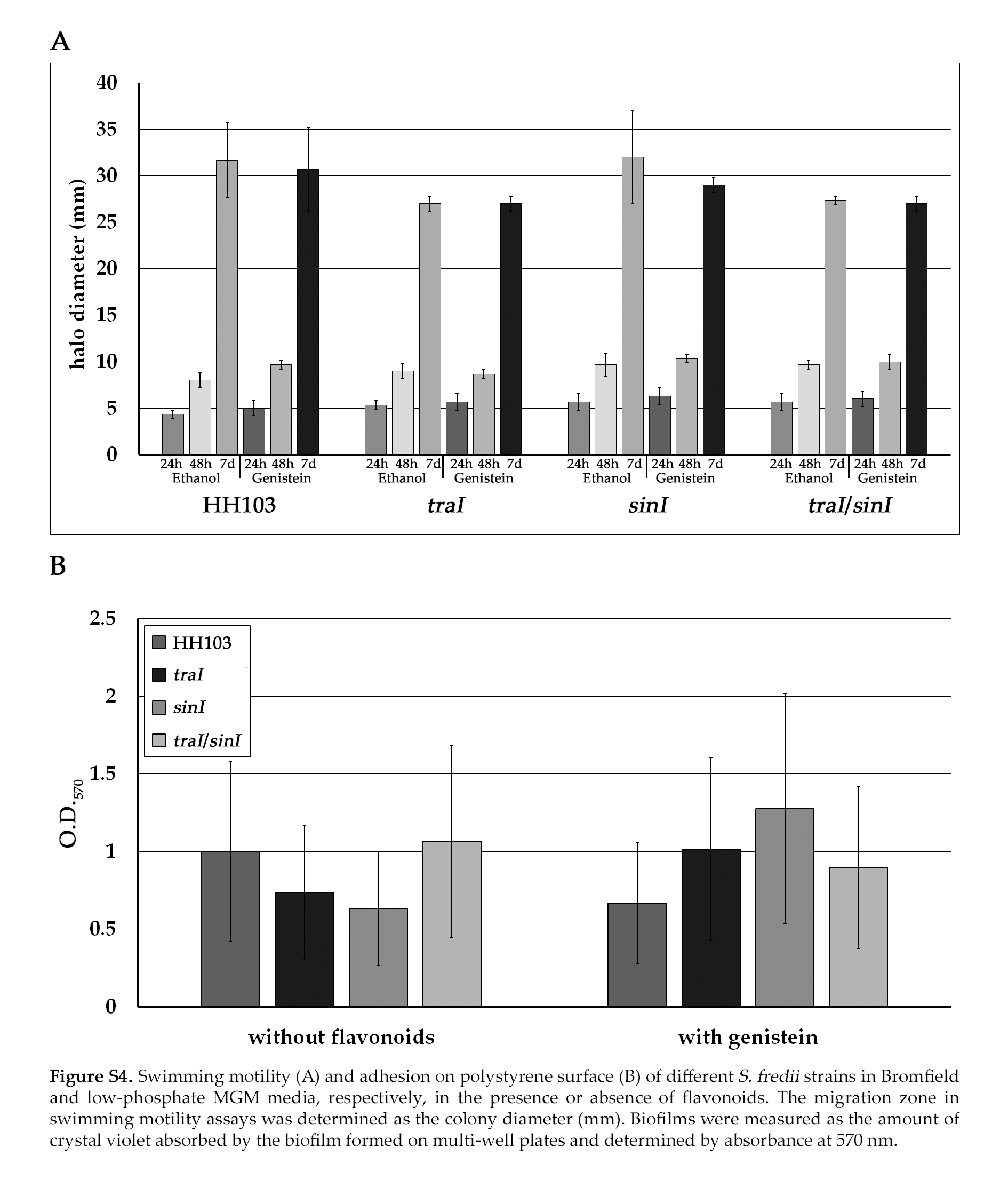

Supplement: Supplementary file 1 [file microorganisms-08-00068-s001.zip › Figure S4 combinado.tif]
